# Endothelial oestrogen–myocardial cyclic guanosine monophosphate axis critically determines angiogenesis and cardiac performance during pressure overload

**DOI:** 10.1093/cvr/cvae202

**Published:** 2024-09-11

**Authors:** Nobuaki Fukuma, Hiroyuki Tokiwa, Genri Numata, Kazutaka Ueda, Pang-Yen Liu, Miyu Tajima, Yu Otsu, Taro Kariya, Yukio Hiroi, James K Liao, Issei Komuro, Eiki Takimoto

**Affiliations:** Department of Cardiovascular Medicine, Graduate School of Medicine, The University of Tokyo, 7-3-1, Hongo, Bunkyo, Tokyo 113-8655, Japan; Division of Cardiology, Department of Medicine, Columbia University Vagelos College of Physicians and Surgeons, New York, NY, USA; Department of Cardiovascular Medicine, Graduate School of Medicine, The University of Tokyo, 7-3-1, Hongo, Bunkyo, Tokyo 113-8655, Japan; Department of Computational Diagnostic Radiology and Preventive Medicine, The University of Tokyo Hospital, Tokyo, Japan; Department of Cardiovascular Medicine, Graduate School of Medicine, The University of Tokyo, 7-3-1, Hongo, Bunkyo, Tokyo 113-8655, Japan; Isotope Science Center, The University of Tokyo, Tokyo 113-0032, Japan; Department of Cardiovascular Medicine, Graduate School of Medicine, The University of Tokyo, 7-3-1, Hongo, Bunkyo, Tokyo 113-8655, Japan; Department of Cardiovascular Medicine, Graduate School of Medicine, The University of Tokyo, 7-3-1, Hongo, Bunkyo, Tokyo 113-8655, Japan; Department of Cardiovascular Medicine, Graduate School of Medicine, The University of Tokyo, 7-3-1, Hongo, Bunkyo, Tokyo 113-8655, Japan; Department of Cardiovascular Medicine, Graduate School of Medicine, The University of Tokyo, 7-3-1, Hongo, Bunkyo, Tokyo 113-8655, Japan; Department of Cardiovascular Medicine, Graduate School of Medicine, The University of Tokyo, 7-3-1, Hongo, Bunkyo, Tokyo 113-8655, Japan; Department of Anesthesiology, Graduate School of Medicine, The University of Tokyo, Tokyo, Japan; Department of Cardiovascular Medicine, National Center for Global Health and Medicine, Tokyo, Japan; Vascular Medicine Research, Brigham and Women’s Hospital, Harvard Medical School, Cambridge, MA, USA; Vascular Medicine Research, Brigham and Women’s Hospital, Harvard Medical School, Cambridge, MA, USA; Department of Medicine, University of Arizona, Tucson, AZ, USA; Department of Cardiovascular Medicine, Graduate School of Medicine, The University of Tokyo, 7-3-1, Hongo, Bunkyo, Tokyo 113-8655, Japan; Department of Cardiovascular Medicine, Graduate School of Medicine, The University of Tokyo, 7-3-1, Hongo, Bunkyo, Tokyo 113-8655, Japan; Division of Cardiology, Department of Medicine, The Johns Hopkins Medical Institutions, 720 Rutland Avenue, Baltimore, MD 21205, USA

**Keywords:** Angiogenesis, Mice, Transgenic, Heart failure, Cyclic GMP, Oestrogen, Non-nuclear signalling

## Abstract

**Aims:**

Oestrogen exerts beneficial cardiovascular effects by binding to specific receptors on various cells to activate nuclear and non-nuclear actions. Oestrogen receptor α (ERα) non-nuclear signalling confers protection against heart failure remodelling, involving myocardial cyclic guanosine monophosphate (cGMP)–cGMP-dependent protein kinase G (PKG) activation; however, its tissue-specific role remains elusive. Herein, we examine the cell type–specific role of ERα non-nuclear signalling in oestrogen-conferred protection against heart failure.

**Methods and results:**

We first assessed the tissue-specific impacts of ERα on the cardiac benefits derived from oestrogen, utilizing endothelial ERα deletion (ERα^f/f^/Tie2^Cre+^) and myocyte ERα deletion (ERα^f/f^/αMHC^Cre+^) female mice. Female mice were ovariectomized and the effect of estradiol (E2) was assessed in hearts exposed to 3 weeks of pressure overload [transverse aortic constriction (TAC)]. E2 failed to improve cardiac function in ERα^f/f^/Tie2^Cre+^ TAC hearts but provided benefits in ERα^f/f^/αMHC^Cre+^ TAC hearts, indicating that endothelial ERα is essential. We next assessed the role of non-nuclear signalling in endothelial cells (ECs), employing animals with endothelial-specific inactivation of ERα non-nuclear signalling (ERα^KI/KI^/Tie2^Cre+^). Female ovariectomized mice were supplemented with E2 and subjected to 3-week TAC. ERα^KI/KI^/Tie2^Cre+^TAC hearts revealed exacerbated cardiac dysfunction and reduced myocardial PKG activity as compared to littermate TAC hearts, which were associated with attenuated myocardial induction of vascular endothelial growth factor (VEGF) and angiogenesis as assessed by CD31-stained capillary density. This phenotype of ERα^KI/KI^/Tie2^Cre+^was rescued by myocardial PKG activation from chronic treatment with a soluble guanylate cyclase (sGC) stimulator. We performed co-culture experiments to determine endothelial–cardiomyocyte interactions. VEGF induction by E2 in cardiac myocytes required a co-existence of intact endothelial ERα signalling in a nitric oxide synthase-dependent manner. On the other hand, VEGF was induced in myocytes directly with an sGC stimulator in the absence of ECs.

**Conclusion:**

An endothelial oestrogen–myocardial cGMP axis stimulates angiogenic response and improves cardiac performance during pressure overload.

## Graphical Abstract

**Graphical Abstract:**
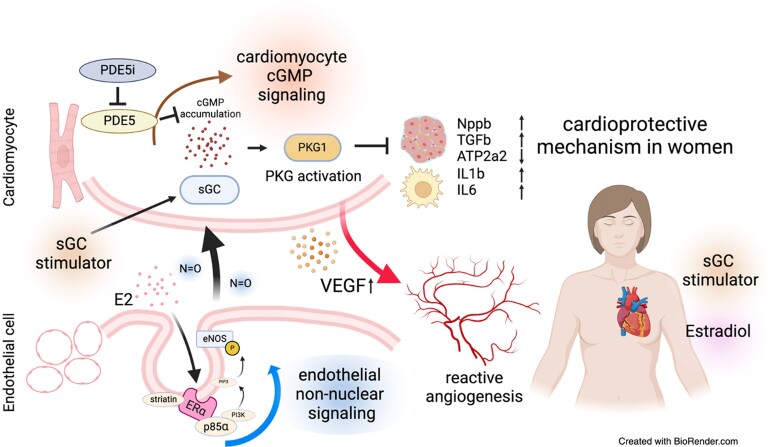

**Time for primary review: 40 days**

## 1. Introduction

Heart failure is a serious condition with significant morbidity and mortality. It affects more than 64 million people worldwide, posing a global social and economic burden.^1,2^ The prevalence of heart failure increases in the older population, particularly that of heart failure with preserved ejection fraction (HFpEF) among women, suggesting the potential involvement of oestrogen deficiency following menopause in the pathogenesis.^3–5^ On the other hand, sex difference in the efficacy of treatment of heart failure has been underscored,^6^ where multiple factors could be potentially involved. The sex-based subgroup analysis in the PARAGON-HF study testing the efficacy of angiotensin receptor–neprilysin inhibitors (ARNi; sacubitril-valsartan, Novartis) for patients with HFpEF identified a more favourable treatment effect in women compared with men.^7,8^ Considering the mechanism of action for ARNi as cyclic guanosine monophosphate (cGMP)-protein kinase G (PKG) activation, the cGMP-PKG pathway might be specifically involved in the pathophysiology of heart failure in women following menopause.

Oestrogen plays critical roles in maintaining cardiovascular homeostasis in women,^9–13^ where diverse effects are mediated by oestrogen receptors, including oestrogen receptor (ER) α, ERβ, G protein-coupled ER (GPR30), and other oestrogen subtypes. Upon binding to ERs, oestrogens exert their effects through nuclear and non-nuclear signalling; the former directly modulates gene transcription and the latter activates kinase signalling pathways with rapid cellular responses.^14^ Among the three receptors, each of which reportedly mediates beneficial cardiovascular effects from oestrogen, ERα has been particularly well-described to couple with endothelial nitric oxide synthase (eNOS)^15–18^ and thus cGMP signalling pathway via its non-nuclear action,^19,20^ playing an essential role in vascular protection, while its nuclear pathway prevents bone demineralization, metabolic derangement of Type 2 diabetes, and atherosclerosis.^21–25^

Utilizing genetically engineered animals with global inactivation of ERα non-nuclear signalling, we recently reported the role of ERα non-nuclear signalling in heart failure, wherein ERα non-nuclear signalling contributes to myocardial PKG activity and cardiac remodelling in female, but not in male, animals.^26^ This study, however, did not explore the tissue-specific role in cardiac protection or its mechanism by ERα non-nuclear signalling. As women are likely to develop microvascular dysfunction with ageing even before presenting with a clinically overt heart failure,^2^ deficient ERα non-nuclear signalling due to oestrogen deprivation might contribute to the pathophysiology of microvascular derangement and heart failure development in women.

In the present study, we determined the role of endothelial ERα non-nuclear signalling in heart failure in women, utilizing a tissue-specific ERα non-nuclear inactivation model. We found that endothelial ERα non-nuclear signalling determines angiogenic responses and cardiac function during pressure overload and that an endothelial oestrogen–myocardial cGMP axis contributes to this regulation.

## 2. Methods

### 2.1 Animals

All animal procedures and the necessary numbers of animals for the experiments were approved by the Institutional Animal Care and Use Committee at the University of Tokyo. In all experiments, animals were euthanized by cervical dislocation under 4.0% of isoflurane anaesthesia. The animal care and experiments were in accordance with the guidelines provided by the NIH.^27^ Animals were housed in cages with a 12 h light–dark cycle in a temperature-controlled laboratory and allowed to access food and water ad libitum. Sildenafil citrate (100 mg/kg/day) was mixed with soft rodent chow (Transgenic Dough Diet; Bio-Serv, Flemington, NJ, USA) for 3 weeks as described previously.^28^ The sGC stimulator (3 mg/kg/day; Riociguat BAY 632521; Bayer, Germany) was diluted in a specific solution (transcutol 10%, cremophor 20%, water 70%) and administered via oral gavage every day for 3 weeks as described previously.^29^

To assess tissue-specific ERα non-nuclear signalling, a novel gene-modified mouse ERα^KI/KI^ generated by Ozgene (Bentley Dc, WA, Australia) was used as described previously.^30^ Briefly, we introduced mutations in exon 4 of ESR1 by replacing amino acid 263R of mouse ERα (corresponding to 259R of human ERα) with alanine, which interrupts the binding between ERα and the PI3 kinase subunit p85α, thereby inhibiting membrane-initiated ERα signalling in response to eNOS. For the tissue-specific gene modification, the cDNA of exon 4–8 from wild-type mouse *ESR1* was sandwiched with two lox sites and integrated into the intron between exon 3 and mutated exon 4. We also generated mice lacking ERα in endothelial cells (ECs) (ERα^f/f^/Tie2^Cre+^) or in cardiomyocytes (CMs) (ERα^f/f^/αMHC^Cre+^) using mice purchased from Jackson Laboratory. All offspring were genotyped by polymerase chain reaction (PCR). These mice were subjected to various experiments and the littermate mice ERα^f/f^/Cre− and ERα^KI/KI^/Tie2^Cre−^ were used as controls.

### 2.2 Ovariectomy and oestrogen replacement

#### 2.2.1 Ovariectomy

Ovariectomy (OVX) was performed in female mice at the age of 6–8 weeks by using a standard bilateral back approach procedure.^31,32^ Briefly, mice were anaesthetized with isoflurane (1.0–1.5% inhalation) and etomidate (E2759; TCI, Tokyo, Japan, intraperitoneal injection, 100 mg/kg) and two small skin incisions (1.5 cm in length) were made on both lateral back sides, immediately caudal to the last rib and 1 cm lateral from the vertebra. The peritoneal muscle was incised and the ovary was identified with oviducts, uterus, and fat tissues. These were then exteriorized with forceps. After a ligation between the ovary and the edge of uterine horn for haemostasis, the ovary was carefully excised.

#### 2.2.2 Oestrogen replacement

One week after OVX, mice were anaesthetized with isoflurane (1.0–1.5% inhalation) and etomidate (intraperitoneal injection, 100 mg/kg), and 60-day time-release estradiol (E2) pellets (0.25 mg, Innovative Research of America, Sarasota, FL, USA) or placebo-containing pellets were subcutaneously implanted on the upper back of the neck as described previously.^33^ This pellet is well-documented to produce physiological levels of E2 in the serum of mice.^34,35^ The uterus size was post-mortally checked to confirm the successful removal of ovaries or the replacement of E2. ERα^KI/KI^/Tie2^Cre+^ animals exhibited a normal uterus morphology, which developed hypertrophy or atrophy depending on E2 levels, similarly to that in wild-type animals (ERα^KI/KI^/Tie2^Cre−^), confirming preserved genomic action (see Supplementary material online, *Figure S1A* and *B*).

### 2.3 Transverse aortic constriction

Mice were subjected to transverse aortic constriction (TAC) or sham surgery (sham) at 8–10 weeks, as described previously.^28^ Briefly, the animals were anaesthetized with isoflurane (1.0–1.5% inhalation) and etomidate (intraperitoneal injection, 100 mg/kg), intubated, and then mechanically ventilated. The transverse aorta was constricted with a 27-gauge needle using a 7-0 Prolene suture. After ensuring a lack of excessive bleeding, the chest was closed, and the animals were allowed to recover from anaesthesia. The animals were euthanized by cervical dislocation under isoflurane anaesthesia (4.0% inhalation) at 3 weeks after TAC. Total heart weight (HW) was measured and normalized to tibial length (TL), and the left ventricular (LV) tissues were harvested. Snap-frozen heart samples were stored at −80°C until analysis.

### 2.4 Echocardiographic study

Transthoracic echocardiography (VEVO2100, 9–18 MHz transducer; Visual Sonics Inc., Toronto, ON, Canada) was performed on conscious mice. M-mode LV end-systolic and end-diastolic dimensions were measured and LV fractional shortenning (%) was calculated as described previously.^33^ These assessments were performed on the day of TAC/sham surgery and at 1 and 3 weeks after TAC/sham surgery by investigators blinded to the genotype and heart condition.

### 2.5 Hemodynamic study using pressure–volume analysis

*In vivo* LV function was assessed by pressure–volume (PV) analysis as described previously.^36^ Mice were anaesthetized with Urethane (94300; Sigma-Aldrich, St. Louis, MO, USA, intraperitoneal injection, 1.2 g/kg) and the LV apex was exposed through an incision between the seventh and eighth ribs. A 1.4-French PV catheter (SPR-839; Millar Instruments; Houston, TX, USA) was inserted from the LV apex and advanced into the LV lumen to lie along the longitudinal axis. The absolute volume was calibrated, and PV data were assessed at the steady state and during the preload reduction phase. Immediately after the hemodynamic measurement, the mice were euthanized with 4.0% isoflurane. Data were analysed using the LabChart application (AD Instruments, Dunedin, New Zealand).

### 2.6 RNA isolation and quantitative RT–PCR

Total RNA was extracted from mouse LV heart samples using TRI reagent® (Molecular Research Center Inc., Cincinnati, OH, USA). The mRNA was reverse transcribed into cDNA using a High Capacity RNA-to-cDNA Kit (Applied Biosystems, Life Technologies, Rockville, MD, USA). The cDNA was amplified using THUNDERBIRD® qPCR Mix (Toyobo Inc., Osaka, Japan), and relative mRNA levels of target genes were measured using a LightCycler 480 (Roche Inc., Basel, Switzerland) with the comparative computed tomography method, as described previously.^37^ Each sample was run in duplicate, and the results were normalized to *Gapdh* mRNA levels. The primer sequences are described in Supplementary material online, *Table S1*.

### 2.7 PKG activity analysis

Colorimetric enzyme immunoassays were performed using the CycLex® cGMP-dependent protein kinase assay kit (MBL International Corporation, Woburn, MA, USA) to determine PKG activity as described previously. Proteins were extracted from whole heart samples as described previously.^36^

### 2.8 Cardiomyocyte isolation and medium VEGFa measurements

CMs were isolated from ovariectomized female ERα^KI/KI^/Tie2^Cre−^ mouse hearts as described previously.^38^ The mice were euthanized by cervical dislocation under isoflurane anaesthesia (4.0% inhalation) and the heart was quickly excised and retroperfused with modified Tyrode’s solution (130 mM NaCl, 5.4 mM KCl, 0.33 mM NaH_2_PO_4_, 0.5 mM MgCl_2_, 22 mM Pure Chemical Corporation, Chuo-ku, Osaka, Japan) through the ascending aorta with 1 mg/mL of collagenase Type 2 (Worthington Biochemical, Lakewood, NJ, USA) and 0.05 mg/mL of protease (Worthington Biochemical). Isolated CMs were incubated at 37°C in a 5% carbon dioxide and 95% air atmosphere. Then, 1 μM sGC stimulator (Riociguat: BAY 632521) diluted in 0.01% dimethyl sulfoxide was administrated and 0.01% of dimethyl sulfoxide was administrated as a vehicle. After 1 day of culture, the cells and media were collected. The protein amount and *Vegfa* expression from CMs were measured. The VEGFa amount in the medium was measured using an enzyme-linked immunosorbent assay kit (MMV00; R&D Systems Inc., Minneapolis, MN, USA) according to the manufacturer’s protocol.

### 2.9 Histology

The heart samples were fixed with 10% formalin and embedded in paraffin. The samples were sliced into 4–5 μm short-axis slices and analysed by immunohistochemistry. Sections at the level of the papillary muscle were stained with Picrosirius red and Fast green for interstitial collagen fraction. Cardiomyocyte cell size was assessed by staining with wheat germ agglutinin (WGA) (Biotin Conjugated Triticum vulgare Lectin; EY, CA, USA). A total of 40 cells per slide were measured and the average value in each sample was calculated. The sections were also stained with rabbit polyclonal antibodies against mouse CD31 (1:200; ab28364; Abcam, Cambridge, UK) to determine the extent of coronary capillary formation as described previously.^39^ EC clusters with lumen morphology were considered capillaries, and all capillaries were counted in at least four fields per section. Capillary density was calculated as the mean number of capillaries per square millimetre. Capillary/cardiomyocyte ratio was calculated as capillary density divided by myocyte density.^40^

### 2.10 EC isolation

ECs were isolated from female mouse brains as previously described.^41^ Briefly, the mice were euthanized by cervical dislocation under isoflurane anaesthesia (4.0% inhalation) and the brains were removed and rinsed in sterilized phosphate buffered saline (PBS). The rinsed brains were minced into small pieces and incubated with papain solution and DNase for 70 min in a 37°C water bath with continuous agitation. The lysates were triturated by aspirating them 10 times each through a 19 G needle and 21 G needle to break up the micro-vessels from the tissue. The isolated ECs were liberated by centrifuging (1360 *g*, 10 min) with 22% bovine serum albumin solution. The cell pellet at the bottom was suspended in the medium with EC growth serum without VEGF (E2759; Sigma-Aldrich) and cultured at 37°C in a 5% carbon dioxide and 95% air atmosphere. After 8–10 days, the ECs proliferated to 90–100% confluency and were co-cultured with wild-type adult female CMs.

### 2.11 Co-culture with ECs and CMs

A total of 5 × 10^4^ ECs of ERα^KI/KI^/Tie2^Cre^ ECswere co-cultured with 2 × 10^5^ cells of CMs isolated from ERα^KI/KI^/Tie2^Cre−^ female mice in the 6.5 mm Transwell® system with 8.0 µm Pore Polycarbonate membrane insert (Corning, Corning, NY, USA) as previously described.^42^ The cells were incubated in phenol red free MEMα (Thermo Fisher Scientific, Waltham, MA, USA) with or without E2 (10^−8^ M) for 24 h at 37°C in a 5% carbon dioxide and 95% air atmosphere. The medium and CMs were collected for the analysis.

### 2.12 EC migration assay

A total of 5 × 10^4^ human umbilical vein endothelial cells (HUVECs)/well were seeded on the upper compartment of the Transwell (8.0 μm pore size, Corning no. 3422) and 2 × 10^5^ cells of isolated CMs from OVX ERα^KI/KI^/Tie2^Cre−^ mice were seeded in each bottom chamber. After 24 h of incubation, the migrated HUVECs were fixed with 4% paraformaldehyde in PBS for 20 min at room temperature, and the cells remaining on the upper compartment side of the Transwell insert membrane were removed with a cotton swab. Then the inserts were washed with PBS with Tween 20 and mounted onto slides with Hoechst (62249; Thermo Fisher Scientific). The migrated cells were counted as previously described.^43^

### 2.13 Statistical analysis

All data were presented as means ± standard error of mean (S.E.M.). The D’Agostino–Pearson normality test was used to determine whether sample distributions were parametric. If the values were normally distributed, the fixed-effects analysis of variance (ANOVA) was used, and one-way or two-way ANOVA was used according to the graph sets. For parametric distribution models, Tukey’s *post hoc* test was applied to evaluate differences between groups. For non-parametric variance models, the Kruskal–Wallis test and Dunn’s *post hoc* multiple comparison tests were used to evaluate statistical significance. Statistical analyses were performed using GraphPad Prism 8 (GraphPad Software, San Diego, CA, USA). *P* values < 0.05 were considered to indicate statistical significance.

## 3. Results

### 3.1 Endothelial ERα, but not myocyte ERα, is critical for oestrogen’s beneficial effects on cardiac function.

**Figure 1:**
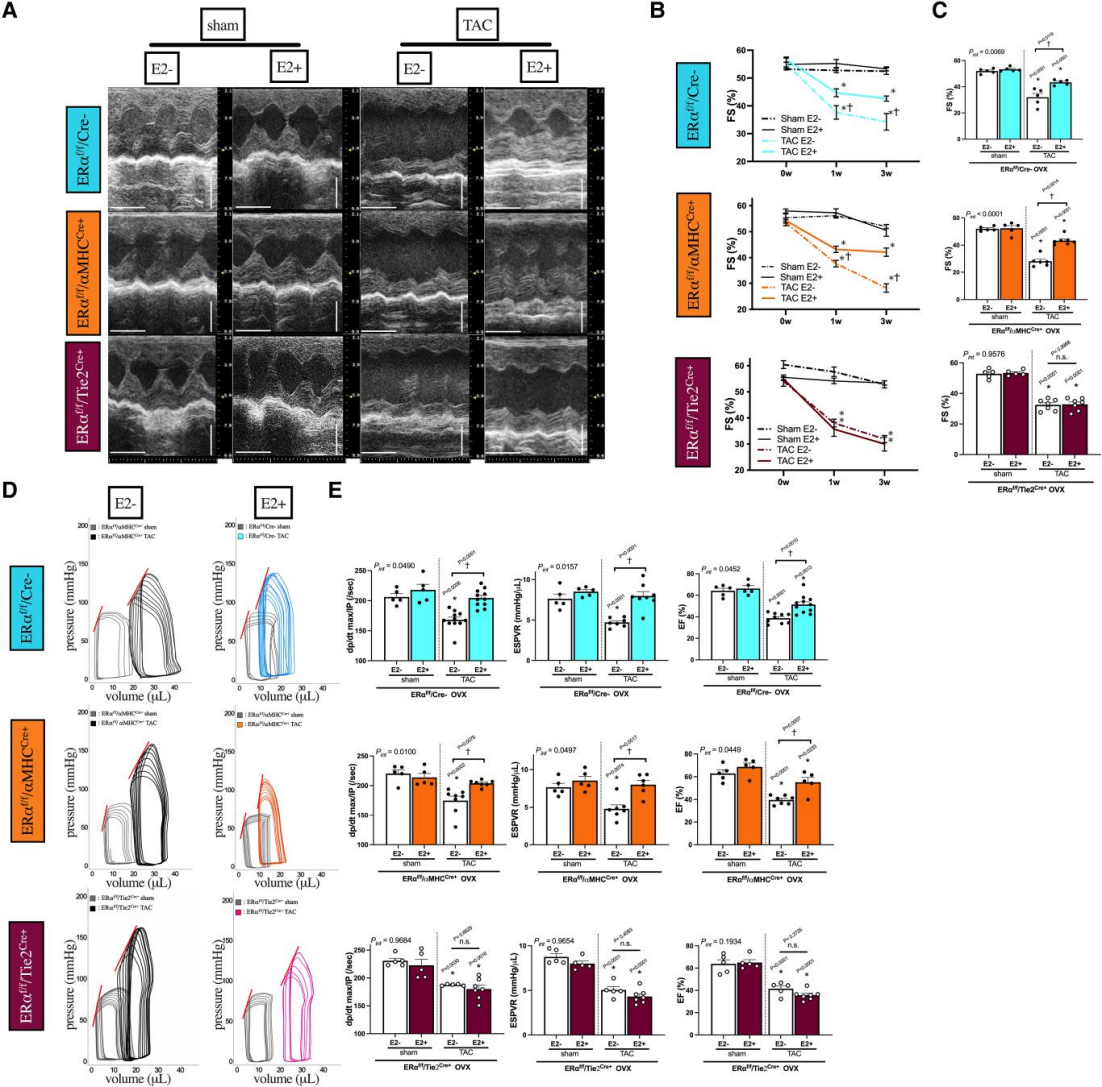
Cardiac phenotype of tissue-specific ERα-knockout mice (OVX ± E2) exposed to LV pressure overload for 3 weeks. (*A*) Representative M mode images of LV by echocardiography at 3 weeks after TAC surgery, time stamp: 100 ms, vertical bar: 2 mm. (*B*) LV FS (%) time course (pre, 1 week, and 3 weeks; *n* = 5–7 per group). (*C*) LV FS (%) at 3 weeks after TAC surgery (*n* = 5–7 per group). (*D*) Representative PV loops of LV at 3 weeks after TAC surgery from ERα^f/f^/Cre−, ERα^f/f^/αMHC^Cre+^, and ERα^f/f^/Tie2^Cre+^ mice. PV loops during pre-load reduction and ESPVR (upper left straight lines) are shown. (*E*) *dP*/*dt* max/IP (/s), ESPVR slope (mmHg/μL), and EF (%) from PV loop analyses at 3 weeks after TAC (*n* = 5–8 per group); *P < 0.05 vs. sham and ^†^*P* < 0.05 vs. TAC E2- were determined by using Tukey’s honest significant difference test following two-way ANOVA; *P*_int_, interaction *P* value as determined by two-way ANOVA; scatter dot plots with bars show individual values and mean ± S.E.M. E2, estradiol; OVX, ovariectomy; *dP*/*dt* max/IP, *dP*/*dt* max normalized to instantaneous pressure; ESPVR, end-systolic pressure–volume relationship; ERα^f/f^/αMHC^Cre+^, mice lacking the ERα in CMs; ERα^f/f^/Tie2^Cre+^, mice lacking the ERα in ECs; ERα^f/f^/Cre−, wild-type littermates.

We first determined the tissue-specific role of ERα by comparing the cardiac benefits of oestrogen in mice lacking ERα in cardiac myocytes (ERα^f/f^/αMHC^Cre+^) with those in mice lacking ERα in ECs (ERα^f/f^/Tie2^Cre+^). The animals were ovariectomized, supplemented with E2, and exposed to 3 weeks of cardiac pressure overload by TAC surgery. The cardiac benefits of E2 were not evident in ERα^f/f^/Tie2^Cre+^ from the assessment of cardiac function assessed with echocardiography (%FS) (*Figure 1A–C*) and invasive PV loop analysis [dP/dt max/IP, end-systolic pressure–volume relationship (ESPVR), EF] (*Figure 1D* and *E*, Supplementary material online, *Figure S2A*), whereas E2 significantly improved cardiac function in ERα^f/f^/αMHC^Cre+^ as in wild types (ERα^f/f^/Cre−).

We previously reported that cGMP-PKG activation with PDE5 inhibition (PDE5i) requires ERα non-nuclear signalling.^26^ In the current study, we also examined the endothelial contribution to this regulation. PDE5i (sildenafil citrate; 100 mg/kg/day) treatment in the presence of E2 further improved %FS by echocardiography and attenuated hypertrophy in ERα^f/f^/αMHC^Cre+^ as in wild types, but not in ERα^f/f^/Tie2^Cre+^ (see Supplementary material online, *Figure S3A* and *B*), which were also consistent with the results obtained from the PV loop analysis, with comparable cardiac afterload [effective arterial elastance (*E*_a_)] and heart rate (HR) between the comparison groups of interest (see Supplementary material online, *Figure S3C* and *D*). These results suggest the important role of endothelial ERα signalling in the cardiac functional benefits from PDE5i.

### 3.2 Endothelial ERα non-nuclear signalling contributes to the beneficial effects of E2 on cardiac function

**Figure 2:**
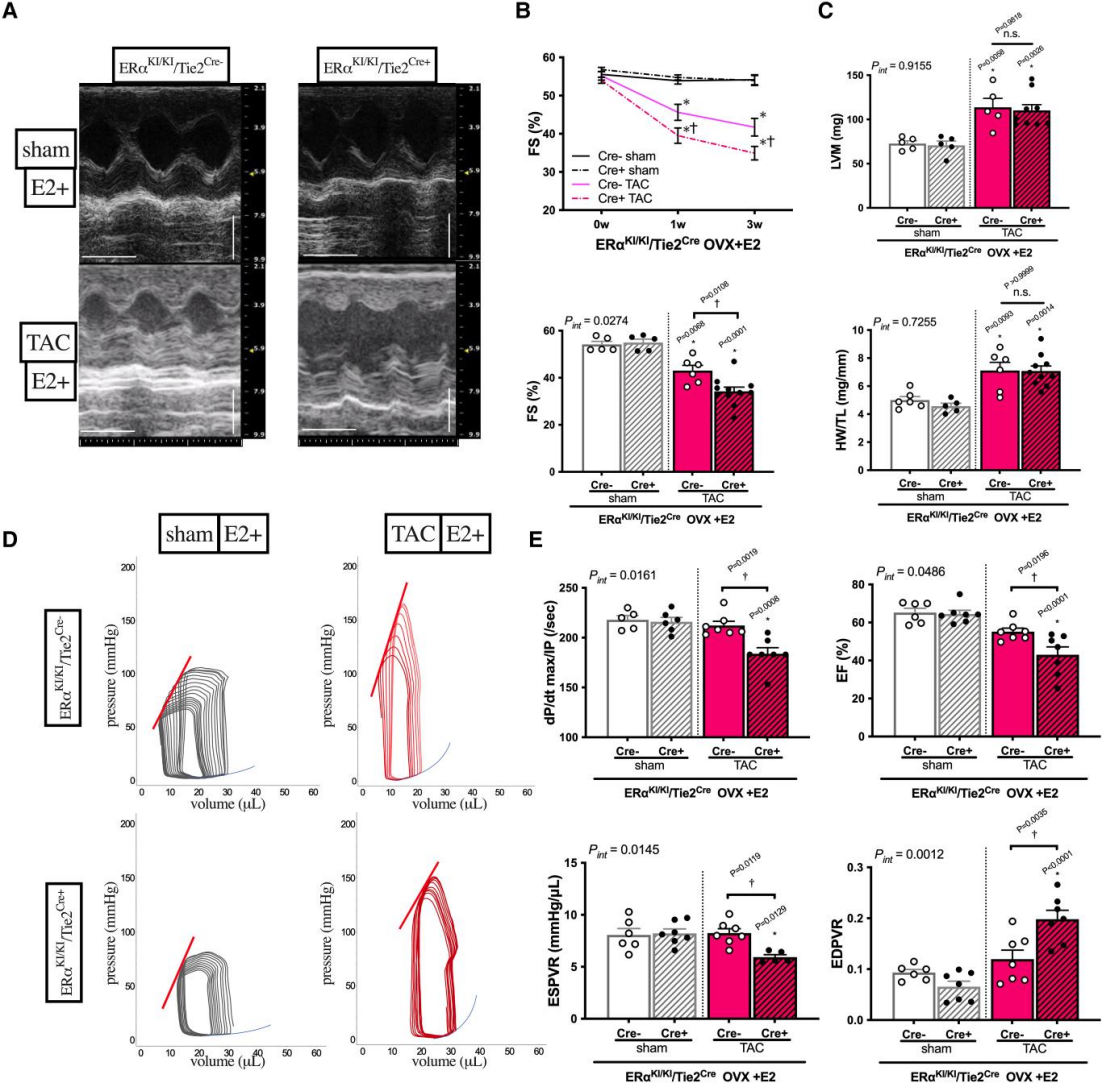
Cardiac phenotype of animals with endothelial-specific ablation of ERα non-nuclear signalling (OVX + E2) exposed to LV pressure overload for 3weeks. (*A*) Representative M mode images of LV by echocardiography at 3 weeks after TAC surgery, time stamp: 100 msec, vertical bar: 2 mm. (*B*) LV FS (%) time course (pre, 1 week, and 3 weeks; upper panel) and LV FS (%) comparison at 3 weeks after TAC surgery (lower panel), time stamp: 100 msec, vertical bar: 2 mm (*n* = 5–11 per group). (*C*) Left ventricular mass (LVM) (mg) by echocardiography (upper panel) and post-mortal HW (mg) normalized to TL (mm) (lower panel) at 3 weeks after TAC surgery (*n* = 5–11 per group). (*D*) Representative PV loops during pre-load reduction (*n* = 5–7 per group) with corresponding ESPVR (upper left straight line) and EDPVR (lower right exponential curve). (*E*) dP/dt max/IP (/sec), EF (%), ESPVR slope (mmHg/μL) and co-efficient β of EDPVR from PV loop analyses at 3 weeks after TAC (*n* = 5–7 per group); *P < 0.05 vs. sham and ^†^*P* < 0.05 vs. Cre− TAC were determined by using Tukey’s HSD test following two-way ANOVA; *P*_int_, interaction *P* value as determined by two-way ANOVA; scatter dot plots with bars show individual values and mean ± S.E.M.; ERα^KI/KI^/Tie2^Cre+^: Cre+, mice lacking ERα non-nuclear signalling in ECs; ERα ^KI/KI^/Tie2^Cre−^: Cre−, wild-type littermates; EDPVR: end-diastolic pressure–volume relationship; other abbreviations as in *Figure 1*.

We next utilized a novel mouse model with selective inactivation of ERα non-nuclear signalling in ECs (ERα^KI/KI^/Tie2^Cre+^) to determine the endothelial ERα-mediated cardioprotection.^30^ Baseline cardiac phenotypes of non-OVX ERα^KI/KI^/Tie2^Cre^ mice were indistinguishable from wild-type littermates,^30^ with none of the three ER subtypes altered at endothelial mRNA expression levels (see Supplementary material online, *Figure S4*). The animals were ovariectomized, supplemented with E2, and then exposed to 3-week TAC. The mutant animals (ERα^KI/KI^/Tie2^Cre+^) exhibited significantly worse cardiac function (lower %FS) after TAC than wild types with E2 supplementation (*Figure 2A* and *B*), and oestrogen’s beneficial effects on cardiac function were diminished in mutant animals (see Supplementary material online, *Figure S5A*). Conversely, cardiac hypertrophy was similarly blunted in wild types and mutant animals with E2 supplementation, as assessed by echocardiographic calculated LV mass [LVM (mg)] and post-mortem measurement of HW normalized to TL (*Figure 2C*, Supplementary material online, *Figure S5B*).

Detailed cardiac functional assessment using PV loop analysis revealed that the dP/dt max/IP, end-systolic pressure volume relationship [ESPVR (mmHg/μL), left upper relationships during preload reduction], and EF were all significantly lower in mutant TAC hearts than in wild-type TAC hearts (*Figure 2D* and *E*). The end-diastolic pressure–volume relationship [EDPVR (mmHg/μL), right lower relationships during preload reduction] was higher in mutant than in wild-type TAC hearts. The HR was comparable (∼600 bpm) among all mouse groups and the TAC mice showed similarly increased LV afterloads compared with those in the sham-operated mice (*E*_a_: 5 mmHg/μL vs. 8–10 mmHg/μL, respectively; Supplementary material online, *Figure S2B*). These results indicate that systolic and diastolic functions were severely impaired after TAC in the absence of endothelial ERα non-nuclear signalling.

The histological analyses of TAC hearts revealed that the reduction of interstitial fibrosis by E2 was significantly blunted in mutant animals, as assessed with Picrosirius red and Fast green staining, despite comparable levels of fibrosis between the genotypes without supplementation of E2 (see Supplementary material online, *Figure S6A* and *B*). Conversely, cell surface area (CSA) of cardiac myocytes, determined from the analyses of WGA-stained sections, was similarly reduced by E2 in either genotype (see Supplementary material online, *Figure S6C* and *D*), suggesting that other ERs might be significantly involved in the anti-hypertrophic effects conferred by oestrogen.

We determined the contribution of ERβ signalling to the anti-hypertrophic effects of E2. Inhibition of ERβ signalling with PHTPP (2.5 mg/kg/every other day) markedly blunted E2-mediated anti-hypertrophic response in ERα^KI/KI^/Tie2^Cre+^ TAC hearts as determined by CSA with minimal impacts on cardiac function (see Supplementary material online, *Figure S7A–C*), suggesting the contribution of ERβ in the anti-hypertrophic mechanism.

### 3.3 Aberrant gene induction and impaired myocardial PKG activation in E2 supplemented ERα^KI/KI^/Tie2^Cre+^ TAC hearts

**Figure 3:**
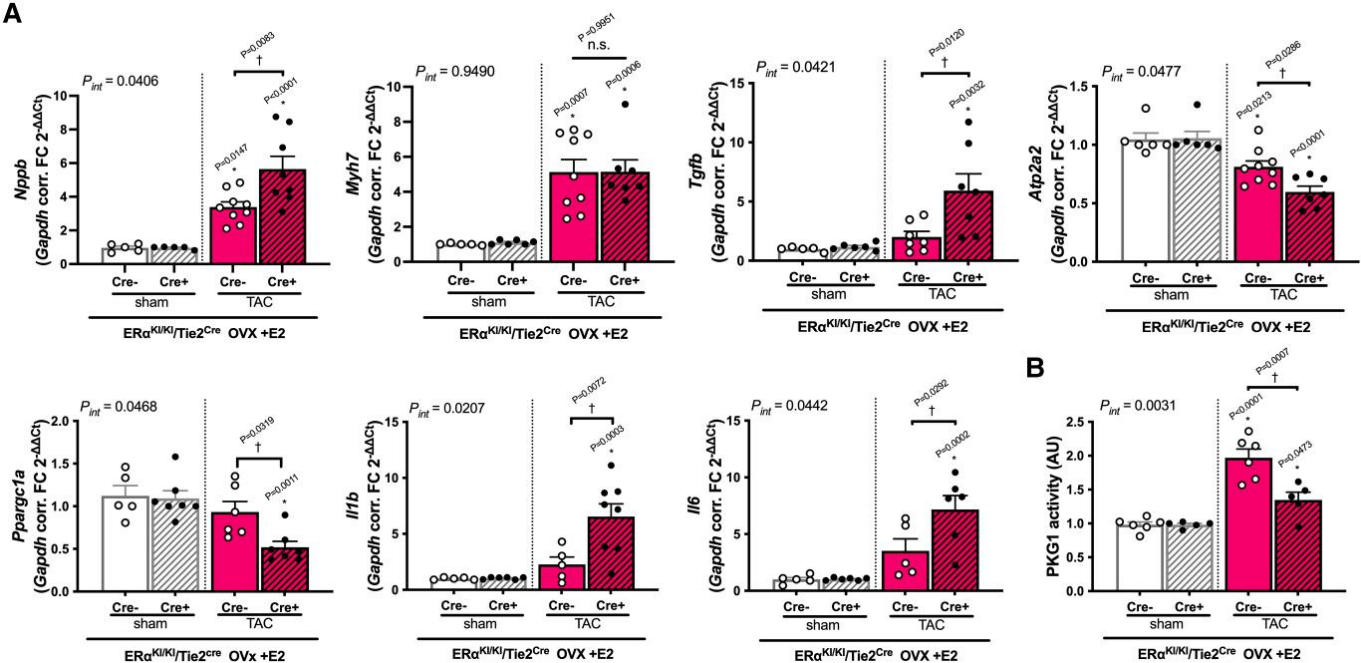
Gene expression and PKG activity in ERα^KI/KI^/Tie2^Cre−^ and ERα^KI/KI^/Tie2^Cre+^ hearts. (*A*) Gene expression in the LV myocardium at 3 weeks after TAC surgery normalized to *Gapdh* (*n* = 5–9 per group). (*B*) PKG1 activity in myocardium (LV free wall) at 3 weeks after TAC surgery (*n* = 5–6 per group); *P < 0.05 vs. sham and ^†^*P* < 0.05 were determined by using Tukey’s HSD test following two-way ANOVA; *P*_int_, interaction *P* value as determined by two-way ANOVA; scatter dot plots with bars show individual values and mean ± S.E.M.; abbreviations are as in *Figures 1* and *2*.

Under the condition of E2 supplementation, mRNA expression levels for *Nppb*, *Tgfb*, *Il1b* , and *Il6* were all significantly higher in ERα^KI/KI^/Tie2^Cre+^ TAC hearts than in wild-type TAC hearts, and those of *Atp2a2* and *Ppargc1a* were significantly reduced in ERα^KI/KI^/Tie2^Cre+^ TAC hearts vs. wild-type TAC hearts (*Figure 3A*), suggesting the development of severe pathological remodelling to pressure overload without endothelial ERα non-nuclear signalling. The *bmhc* (*Myh7*) was similarly increased by TAC in both genotypes, consistent with the comparable hypertrophic response. We assessed myocardial PKG activity in these E2-supplemented hearts in light of the oestrogen-cGMP axis. Myocardial PKG activity was significantly reduced in mutant TAC hearts compared with that in wild-type TAC hearts, but there was no difference in sham controls between the genotypes (*Figure 3B*), indicating the pivotal role of endothelial ERα non-nuclear signalling in PKG activation in female hearts after pressure overload.

### 3.4 Inactivation of endothelial ERα non-nuclear signalling abrogates angiogenic responses to TAC

**Figure 4:**
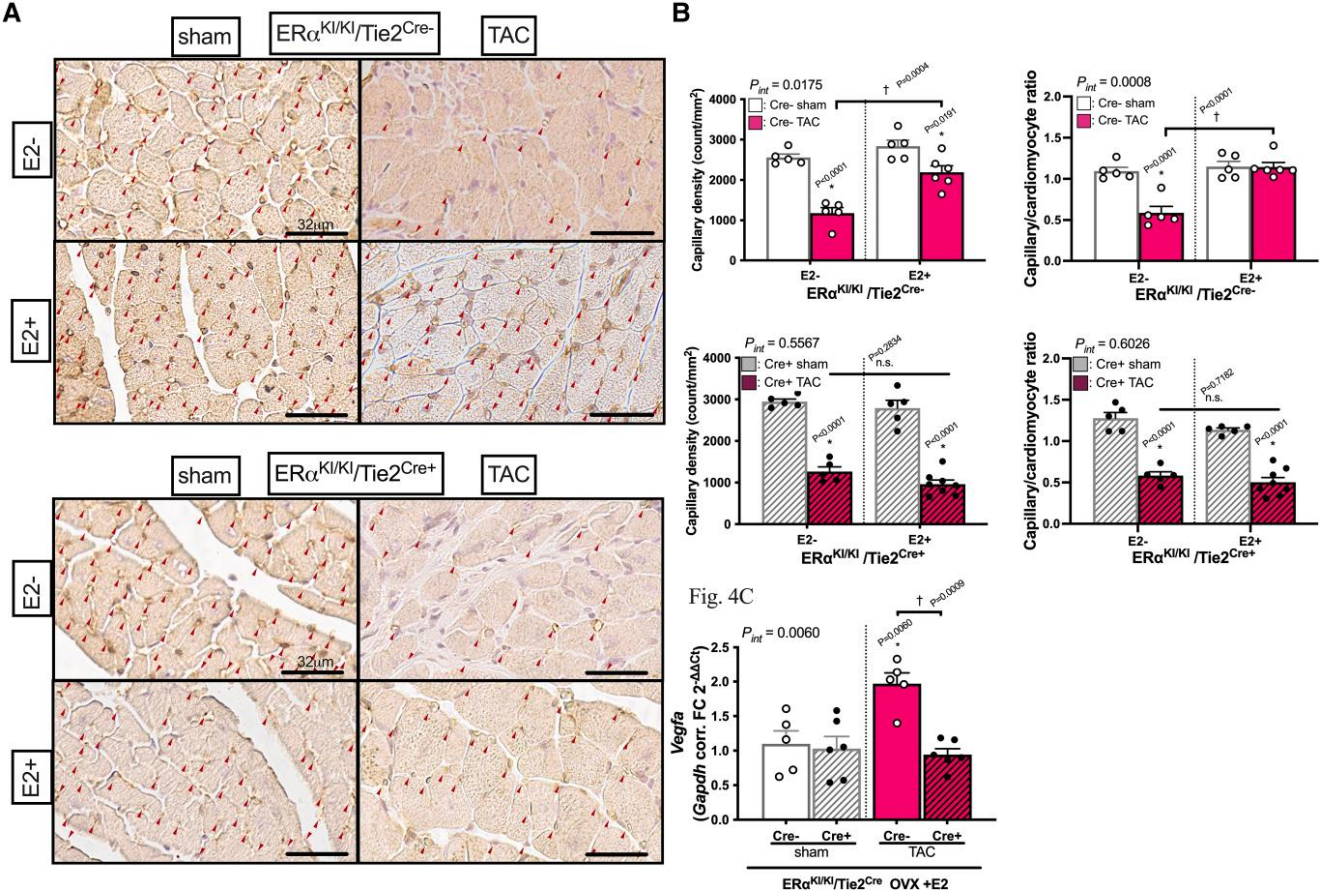
Angiogenesis in ERα^KI/KI^/Tie2^Cre−^ and ERα^KI/KI^/Tie2^Cre+^ OVX hearts exposed to 3-week pressure overload with or without E2 supplementation. (*A*) Representative histological images of left ventricles stained with CD31 at 3 weeks after TAC surgery (arrow heads indicate CD31-positive micro-vessels; scale bar: 32 μm). (*B*) Quantification results of capillary density (counts/mm^2^) at 3 weeks after TAC surgery in ERα^KI/KI^/Tie2^Cre−^ and ERα^KI/KI^/Tie2^Cre+^ hearts (*n* = 5–6 per group). (*C*) Myocardial expression of *Vegfa* at 3 weeks after TAC surgery with E2 supplementation (*n* = 5–6 per group); *P < 0.05 vs. sham and ^†^*P* < 0.05 were determined by using Tukey’s HSD test following two-way ANOVA; *P*_int_, interaction *P* value as determined by two-way ANOVA; scatter dot plots with bars show individual values and mean ± S.E.M.; abbreviations are as in *Figures 1* and *2*.

As cardiac function was severely impaired by TAC in mutant hearts despite similar degree of hypertrophy development to wild-type TAC hearts, we examined angiogenic responses in the LV myocardium, which is one of the key mechanisms for adaptation to chronic loading stress. Capillary density, assessed by CD31 staining, was significantly reduced by TAC without oestrogen in wild-type mice but was maintained in the presence of oestrogen. By contrast, such angiogenic responses to oestrogen after TAC were not observed in mutant hearts (*Figure 4A* and *B*). In the presence of oestrogen, mRNA upregulation of *Vegfa*, a potent angiogenic factor secreted primarily from CMs, was markedly increased by TAC in wild-type hearts, but was virtually lost in mutant hearts (*Figure 4C*). These results suggest that endothelial ERα non-nuclear signalling is critically involved in the angiogenic response during pressure overload in female hearts, contributing to maintaining micro-circulation.

### 3.5 Cardiomyocyte-derived VEGFa that is induced by cGMP-PKG critically affects angiogenesis after TAC

**Figure 5:**
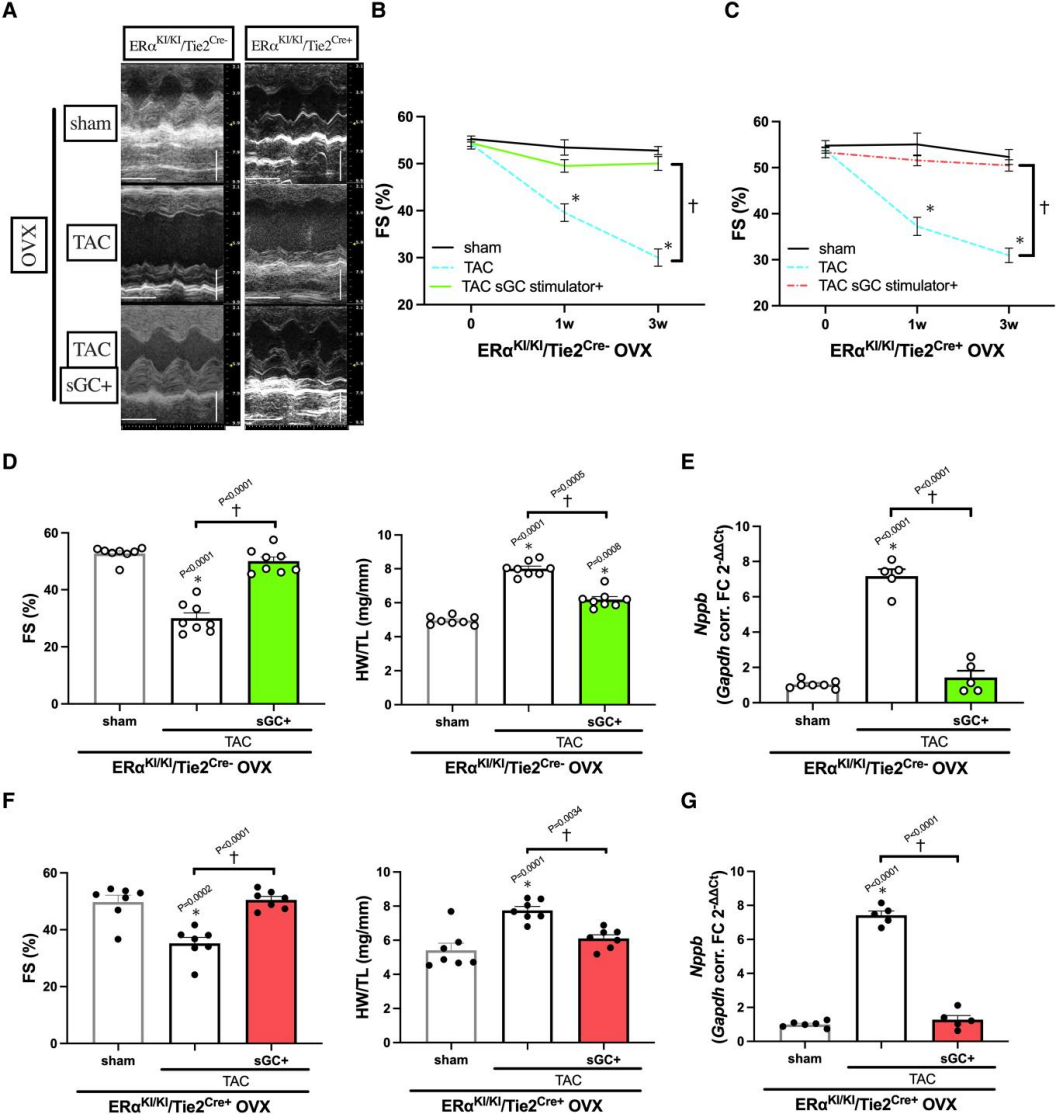
The effect of sGC stimulation on ERα^KI/KI^/Tie2^Cre−^ and ERα^KI/KI^/Tie2^Cre+^ OVX hearts subjected to pressure overload. (*A*) Representative M mode images of LV by echocardiography at 3 weeks after TAC surgery, time stamp: 100 msec, vertical bar: 2 mm. (*B*) LV FS (%) time course for ERα^KI/KI^/Tie2^Cre−^ mice after TAC surgery (pre, 1 week, and 3 weeks; *n* = 8 per group). (*C*) LV FS (%) time course for ERα^KI/KI^/Tie2^Cre+^ mice after TAC surgery (pre, 1 week, and 3 weeks; *n* = 7 per group). (*D*) LV FS (%) and HW (mg) normalized to TL (mm) of ERα^KI/KI^/Tie2^Cre−^ mice at 3 weeks after TAC surgery (*n* = 8 per group). (*E*) *Nppb* expression in the LV myocardium of ERα^KI/KI^/Tie2^Cre−^ mice at 3 weeks after TAC surgery (*n* = 5–7 per group). (*F*) LV FS (%) and HW (mg) normalized to TL (mm) of ERα^KI/KI^/Tie2^Cre+^ mice at 3 weeks after TAC surgery (*n* = 7 per group). (*G*) *Nppb* expression in the LV myocardium of ERα^KI/KI^/Tie2^Cre+^ mice at 3 weeks after TAC surgery (*n* = 7 per group); *P < 0.05 vs. sham and ^†^*P* < 0.05 were determined by using Tukey's HSD test following one-way ANOVA; scatter dot plots with bars show individual values and mean ± S.E.M.; abbreviations are as in *Figures 1* and *2*.

**Figure 6:**
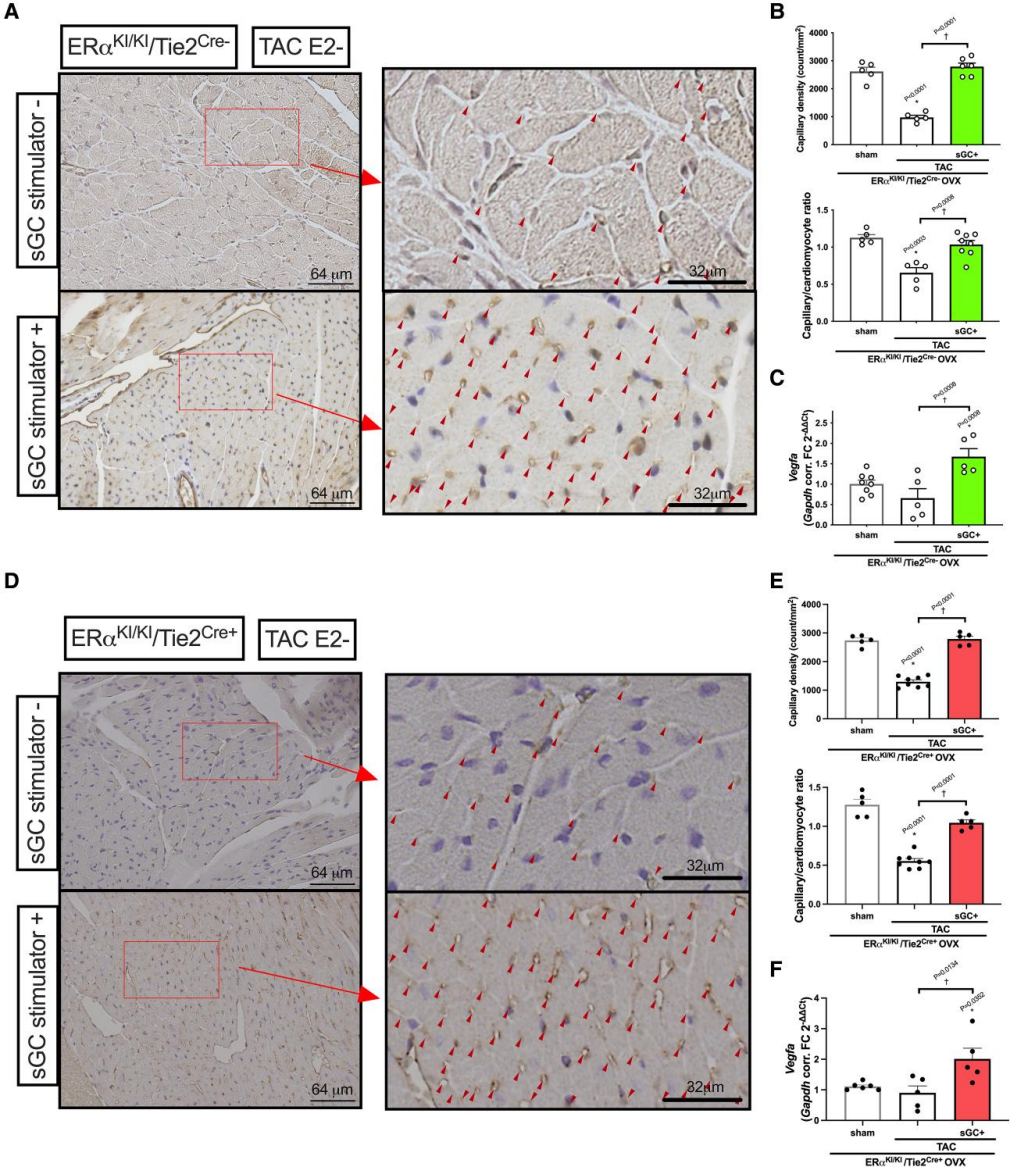
Angiogenesis in E2-deprived ERα^KI/KI^/Tie2^Cre^ 3-week TAC hearts treated with an sGC stimulator. (*A*) Representative histological images of ERα^KI/KI^/Tie2^Cre−^ left ventricle stained with CD31 at 3 weeks after TAC surgery with concomitant treatment with sGC stimulation (arrow heads indicate CD31-positive micro-vessels; scale bars: 64 μm for left panels and 32 μm for right panels). (*B*) Quantification results on capillary density (counts/mm^2^) (upper panel) and capillary/cardiomyocyte ratio (lower panel) at 3 weeks after TAC surgery for OVX ERα^KI/KI^/Tie2^Cre−^ mice (*n* = 5–6 per group). (*C*) *Vegfa* expression in the LV myocardium at 3 weeks after TAC surgery with concomitant treatment with sGC stimulation in OVX ERα^KI/KI^/Tie2^Cre−^ mice (*n* = 5–8 per group). (*D*) Representative histological images of ERα^KI/KI^/Tie2^Cre−^ left ventricle stained with CD31 at 3 weeks after TAC surgery with concomitant treatment with sGC stimulation (arrow heads indicate CD31-positive micro-vessels; scale bars: 64 μm for left panels and 32 μm for right panels). (*E*) Quantification results on capillary density (counts/mm^2^) (upper panel) and capillary/cardiomyocyte ratio (lower panel) at 3 weeks after TAC surgery for OVX ERα^KI/KI^/Tie2^Cre+^ mice (*n* = 5–8 per group). (*F*) *Vegfa* expression in the LV myocardium at 3 weeks after TAC surgery with concomitant treatment with sGC stimulation in OVX ERα^KI/KI^/Tie2^Cre+^ mice (*n* = 5–6 per group); *P < 0.05 vs. sham and ^†^*P* < 0.05 were determined by using Tukey’s HSD test following one-way ANOVA; scatter dot plots with bars show individual values and mean ± S.E.M.; abbreviations are as in *Figures 1* and *2*.

**Figure 7:**
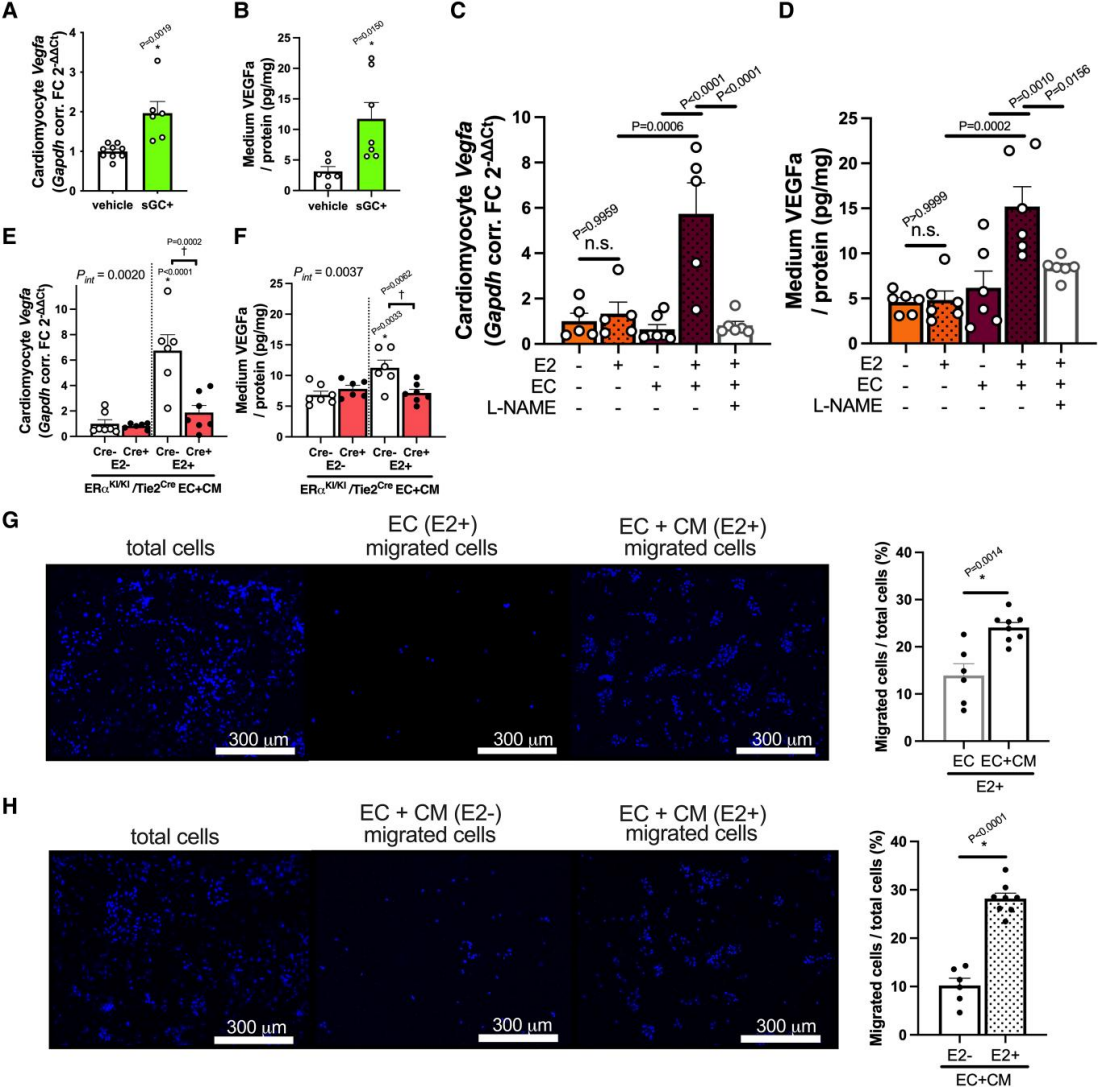
Interactive mechanism between ECs and CMs via oestrogen-cGMP signalling pathways. *Vegfa* expression in cultured CMs isolated from wild-type adult mice in the presence of an sGC stimulator (stimulated for 24 h) (*A*) and the amount of VEGFa protein (normalized to cardiomyocyte protein amount) in the culture medium (*B*). *Vegfa* expression in wild-type CMs co-cultured with wild-type (ERα^KI/KI^/Tie2^Cre−^) ECs in the presence or absence of E2/L-NAME (*C*) and the amount of VEGFa protein (normalized to cardiomyocyte protein amount) in the culture medium (*D*). *Vegfa* expression in wild-type CMs co-cultured with ERα^KI/KI^/Tie2^Cre−^ (Cre−) or ERα^KI/KI^/Tie2^Cre+^ (Cre+) ECs in the presence or absence of E2 (*E*) and the protein amount of VEGFa (normalized cardiomyocyte protein amount) in the culture medium (*F*). (*G*) Representative images of E2-stimulated HUVEC (EC) migration with or without co-culture of wild-type (ERα^KI/KI^/Tie2^Cre−^) CMs (left panels). The ratio of migrated ECs to total seeded ECs (%) shown in the right bar graphs (*n* = 6–8 wells per group; scale bar: 300 μm). (*H*) Representative images of HUVEC (EC) migration in the presence or absence of E2 when EC and wild-type CMs were co-cultured. The ratio of migrated ECs to total seeded ECs (%) shown in the right bar graphs (*n* = 6–8 wells per group; scale bar: 300 μm); *P < 0.05 in *Figure 7A, B, G,* and *H* was determined by using Student’s *t*-test. *P* values in *Figure 7C* and *D* were determined by using Tukey’s HSD test following one-way ANOVA. *P < 0.05 vs. E2- and ^†^*P* < 0.05 in *Figure 7E* and *F* were determined by using Tukey’s HSD test following two-way ANOVA; *P*_int_, interaction *P* value as determined by two-way ANOVA; scatter dot plots with bars show individual values and mean ± S.E.M. HUVEC, human umbilical vein endothelial cells; ECs, endothelial cells; CMs, cardiomyocytes; other abbreviations are as in *Figures 1* and *2*.

We next tested if the cGMP-PKG pathway, downstream of endothelial ERα non-nuclear signalling, directly regulates angiogenesis in oestrogen-deprived TAC hearts treated with sGC-cGMP stimulation. The sGC stimulation activated myocardial cGMP-PKG and improved cardiac function (*Figure 5A–D* and *F*) with attenuated *Nppb* gene expression (*Figure 5E* and *G*) during pressure overload even in the absence of oestrogen both in ERα^KI/KI^/Tie2^Cre−^ and in ERα^KI/KI^/Tie2^Cre+^ mice, consistent with our prior work.^26^ Importantly, the hearts treated with sGC stimulation revealed increased capillary density regardless of endothelial ERα^KI/KI^ gene mutations (*Figure 6A, B, D,* and *E*), associated with mRNA upregulation of *Vegfa* (*Figure 6C* and *F*), suggesting angiogenic control via direct myocardial cGMP-PKG activation. We then tested if CMs are involved in this regulation, given that these cells are a significant source of VEGF secretion. In cultured CMs isolated from ERα^KI/KI^/Tie2^Cre−^ mouse hearts, overnight sGC stimulation upregulated *Vegfa* expression and also increased the medium VEGFa concentration, indicating the involvement of CMs in *Vegfa* expression induced via sGC-cGMP signalling (*Figure 7A* and *B*).

### 3.6 E2 induces myocyte VEGF production via endothelial–cardiomyocyte cross-talk that is mediated by endothelial ERα non-nuclear signalling

To further elucidate the interactive mechanism between ECs and CMs, ECs isolated from ERα^KI/KI^/Tie2^Cre+^ mice or wild-type littermates were co-cultured with adult CMs from female wild-type mice, and the effects of E2 were examined. *Vegfa* expression in CMs, as well as VEGFa amounts in the culture media, was markedly increased by E2 when CMs and wild-type ECs were co-cultured, whereas oestrogen did not have a significant impact on either *Vegfa* expression in CMs or VEGFa amount in the culture media when CMs were cultured alone (*Figure 7C* and *D*). Importantly, such VEGF production was abrogated by NOS inhibition (*Figure 7C* and *D*) and markedly reduced when CMs were co-cultured with the ECs from ERα^KI/KI^/Tie2^Cre+^ mice (*Figure 7E* and *F*). We further performed migration assay of ECs utilizing HUVECs for determining the functional impact of CM-released VEGF on ECs in this co-culture system (*Figure 7G* and *H*). E2-induced EC migration was enhanced when ECs were co-cultured with CMs, indicating the activation of endothelial VEGF signalling via the interaction with CMs. These results support the mechanistic link between ECs and CMs via ERα non-nuclear signalling in ECs.

### 3.7 Endothelial ERα non-nuclear signalling does not have significant impact in male hearts under the condition without hormonal intervention

Lastly, we also examined the role of endothelial ERα non-nuclear signalling in males under the condition without any hormonal intervention. Male ERα^KI/KI^/Tie2^Cre+^ hearts exposed to 3-week TAC revealed LV remodelling comparable to wild-type littermates, as assessed by echocardiographic evaluation [FS (%) and LVM (mg)], post-mortal HW, and myocardial foetal gene expression analyses, suggesting that endothelial ERα non-nuclear signalling might not play a significant role in LV remodelling with intact sex hormone levels (see Supplementary material online, *Figure S8A–C*).

## 4. Discussion

We previously reported the role for ERα non-nuclear signalling and its indispensability in the therapeutic efficacy of PDE5i in female hearts; however, its tissue-specific role was not explored.^26,33^ In the current study, we employed a novel mouse model and further identified endothelial ERα non-nuclear signalling as a key regulator of cardiac function by activating myocardial cGMP–PKG and inducing angiogenesis during pressure overload. Our data suggest that oestrogen deficiency could contribute to the pathogenesis of heart failure via a mechanism involving insufficient myocardial cGMP–PKG activation coupled with impaired angiogenesis. Importantly, cGMP–PKG activation with sGC stimulation stimulates angiogenesis in such oestrogen-deficient hearts and ameliorates heart failure, which might form an additional mechanistic basis for the therapeutic use of sGC stimulators.

We demonstrated the pivotal role of endothelial ERα non-nuclear signalling in the intrinsic myocardial cGMP-PKG activation during cardiac pressure overload in female hearts. The prior study by Menazza *et al*.^19^ also supports the role of ECs in the cardiac benefits of [oestrogen dendrimer conjugates (EDCs)]. The authors demonstrated that the infarct size reduction by EDCs following ischemia reperfusion (I/R) is abrogated in endothelial-specific ERα-knockout mice. This regulation, however, involves *S*-nitrosylation and not cGMP-PKG pathway, as they observe that EDC-mediated cardiac protection is associated with *S*-nitrosylation of proteins, and is not blocked by sGC inhibition but by NOS-inhibition. Therefore, either mechanisms might be at work for cardiac protection depending on the context (i.e. chronic vs. acute).

In our prior work, we reported that oestrogen non-nuclear signalling also exists inCMs, which enhances NO–cGMP–PKG signalling via ERα.^27^ The present work, however, revealed that the effects of PDE5i on cardiac-specific ERα-knockout animals were similar to those on wild-type animals (see Supplementary material online, *Figure S3*), indicating the predominant contribution of NO from intact endothelial ERα non-nuclear signalling to myocardial cGMP–PKG activity, which is coupled with VEGF production to angiogenesis and thus improving cardiac function. The *in vitro* culture experiment results of the minimal production of VEGFa by E2 in wild-type CM indicate that E2-enhanced cGMP–PKG signalling in CMs alone might not be potent enough to induce VEGF production and thus angiogenesis (*Figure 7C* and *D*). Stress-responsive production of VEGF in CMs and thereby the induction of angiogenesis is crucial for the adaptive hypertrophic response to pressure overload.^44,45^ Sufficient delivery of nutrients and oxygen could contribute to the improvement of cardiac function, and an increase in the capillary mass itself could boost the endothelium-derived paracrine system leading to adaptation.

In contrast to cardiac functional effects, the anti-hypertrophic effects of E2 were unexpectedly unaffected in ERα^KI/KI^/Tie2^Cre+^ TAC hearts, which were abrogated by the additional inhibition of ERβ signalling. These indicate that the anti-hypertrophic effects of E2 might be predominantly mediated by intact ERβ signalling activation than endothelial NO-activated cGMP-PKG signalling, and the physiological impacts from these may depend on how cGMP-PKG is activated due to the sub-cellular compartmentalized regulation.^46^ Our results are in line with prior publication by others. Pedram *et al*.^47^ reported that ERβ signalling significantly contributes to the anti-hypertrophic effects of oestrogen via histone deacetylase regulation. ERβ activation also reportedly phosphorylates eNOS and produces NO independently from the ERα pathway.^48^ Given E2-stimulated NOS activity in mutant ECs is reduced by 50% vs. wild types,^30^ ERβ-NOS axis does not compensate for the loss of ERα non-nuclear signalling. Conversely, accumulating evidence has shown that G protein-coupled oestrogen receptor 1 (GPR30) mediates E2-conferred cardiac protection against maladaptive cardiac remodelling via non-nuclear mechanisms.^49,50^ We have not determined the contribution of the GPR30-mediated pathway in the current study. However, it is very likely that all three oestrogenic pathways including ERα, ERβ, and GPR30 might contribute to cardiac protection against maladaptive cardiac remodelling to a varying extent depending on disease stages. Also, complex interactions may exist among these pathways.^51^

We observed that *Il1b* and *Il6* were upregulated in mutant TAC hearts vs. wild-type TAC hearts under E2-supplemented conditions. Oestrogens have been reported to reduce inflammation by the interfering NF-kB pathway through genomic and non-genomic mechanisms in immune cells.^52^ Given that ERα non-nuclear signalling is disrupted in ECs with immune cells being intact in our mutant animals, the augmented inflammation despite oestrogen presence in our mutant TAC hearts would be attributable to the dysfunctional endothelium with the insufficient NO-cGMP signalling pathway. As NO reportedly decreases the expression of monocyte chemoattractant protein 1, vascular cell adhesion molecule 1, and subsequent adhesion of immune cells, dysfunction of this pathway leads to facilitation of the engraftment of immune cells, triggering fibrosis and a vicious cycle of inflammatory response. Insufficient myocardial perfusion due to microvascular rarefaction was also reported to induce cardiac remodelling.^53^ Microvascular rarefaction may contribute to the more inflamed condition in mutant hearts.

Our mutant TAC model also revealed pronounced diastolic dysfunction, as assessed with EDPVR from PV loop analysis (*Figure 2E*). This could be attributed to microvascular rarefaction, augmented inflammation, myocardial fibrosis, and impaired myocardial PKG activity in mutant hearts. These are all reported to impair cardiac diastolic function,^54–57^ highlighting the pivotal role of endothelial ERα non-nuclear signalling in the regulation of diastolic function in female cardiac pathologies.

Oestrogen treatment reportedly confers cardioprotection in male rodent models. We have not tested whether such additional E2 administration provides benefits via the ERα-cGMP-PKG mechanism as in males; however, we found that ERα non-nuclear signalling had minimal impact on cardiac pathologies in males under the condition without hormonal intervention. On the other hand, androgens have been shown to induce angiogenesis via the VEGF-Akt-eNOS pathway in male vascular models.^58,59^ It would be reasonable to speculate that the androgen-cGMP-PKG axis could also play a significant clinical role in heart failure in males.

Our study has the following limitations. First, we performed OVX in mice at 6–8 weeks of age to assess the pathophysiology of oestrogen deprivation, mimicking post-menopausal status, as the model has been established for this purpose. However, utilizing an older female cohort would provide insights better related to the actual patient situation involving the effect of ageing. Second, we did not determine serum oestrogen levels in the current study. The E2 pellets employed here are designed to provide a stable physiological serum concentration of E2 without the estrous cycle, and the dose we chose has been widely used in prior publications determining oestrogenic effects in the cardiovascular system in mice.^31,33–35^ Third, we did not measure the aortic pressure gradient by echocardiography to assess the severity of TAC.^60^ Instead, we assessed *E*_a_ from haemodynamic analysis, one of the most accurate and reliable indices of LV afterload,^40^ and the results revealed a comparable increase of *E*_a_ by TAC in either genotype. Lastly, we did not explore potential angiogenesis-inducing pathways other than cGMP-PKG, which might include the PI3K-Akt signalling pathway.^61^ Future studies are warranted for clarification.

**Translational perspective**

- Targeting endothelial oestrogen receptor α non-nuclear signalling might be a new strategy to treat heart failure through nitric oxide (NO)-soluble guanylate cyclase (sGC)-protein kinase G activation without oestrogen’s various unfavourable effects.
- Reactive angiogenesis is one of the cardioprotective mechanisms via sGC stimulation that directly stimulates cardiomyocytes (CMs) regardless of oestrogen status or endothelial cell status.
- Given oestrogen’s critical cyclic guanosine monophosphate (cGMP) augmenting in CMs via endothelial functions, our findings may provide the potential mechanistic background of sex differences in cGMP-related treatments for heart failure.

## 5. Conclusion

Our study demonstrated the pivotal role of endothelial ERα non-nuclear signalling in the cardiac functional benefits of oestrogen mediated by an ERα non-nuclear signalling cGMP-PKG axis, wherein reactive angiogenesis was induced by bi-directional interaction between endothelial ERα non-nuclear signalling and cardiac myocyte cGMP signalling. This study also clarified one of the cardioprotective mechanisms of sGC stimulation: Myocyte cGMP activation by sGC stimulation could compensate for this axis, together with other cGMP-PKG-derived cardiac benefits regardless of oestrogen status or EC status. Accordingly, these findings form another mechanistic basis for the use of an sGC stimulator for the treatment of heart failure. From the point of view of oestrogens, targeting endothelial ERα non-nuclear signalling might be a promising heart failure treatment without oestrogen’s unfavourable side effects caused by nuclear signalling. Our findings also provide the mechanistic insights into our understanding of sex differences in cGMP-related treatments for heart failure.

## Supplementary Material

cvae202_Supplementary_Data

## Data Availability

All data presented in this article are available in the main article and in its online Supplementary materials.
