# Supplementary material for: Endothelial oestrogen–myocardial cyclic guanosine monophosphate axis critically determines angiogenesis and cardiac performance during pressure overload
Source: Cardiovasc Res. 2024 Sep 11;120(15):1884–97. doi: 10.1093/cvr/cvae202 (PMC11630045; doi:10.1093/cvr/cvae202)
Supplement: cvae202_Supplementary_Data [file cvae202_supplementary_data.pdf]

Endothelial estrogen - myocardial cGMP axis critically determines angiogenesis and cardiac performance during pressure-overload

Fukuma et al.: Endothelial ERα non-nuclear signaling - myocardial cGMP axis determines angiogenesis and cardiac performance

Nobuaki Fukuma, M.D. PhD.[a, d*], Hiroyuki Tokiwa, M.D. PhD.[a, g*], Genri Numata, M.D. PhD.[a, h*], Kazutaka Ueda, M.D. PhD.[a], Pangyen Liu, M.D. PhD.[a], Miyu Tajima, M.D. PhD.[a], Yu Otsu, M.D., PhD.[a], Taro Kariya, M.D., PhD.[a, i], Yukio Hiroi M.D. PhD.[c, e], James K. Liao M.D.[e, f], Issei Komuro, M.D., PhD.[a], Eiki Takimoto, M.D. PhD.[a, b]

[a] Department of Cardiovascular Medicine, Graduate School of Medicine, The University of Tokyo, 113-8655, Japan

[b] Division of Cardiology, Department of Medicine, The Johns Hopkins Medical Institutions, 720 Rutland Avenue, Baltimore, MD 21205, USA

[c] Department of Cardiovascular Medicine, National Center for Global Health and Medicine, Tokyo, Japan

[d] Division of Cardiology, Department of Medicine, Columbia University Vagelos College of Physicians and Surgeons, New York, NY, USA.

[e] Vascular Medicine Research, Brigham and Women's Hospital and Harvard Medical School, Cambridge, MA, USA

[f] Department of Medicine, University of Arizona, Tucson, AZ, USA

[g] Department of Computational Diagnostic Radiology and Preventive Medicine, The University of Tokyo Hospital, Tokyo, Japan

[h] Isotope Science Center, The University of Tokyo, Tokyo 113-0032, Japan

[i] Department of Anesthesiology, Graduate School of Medicine, The University of Tokyo, Tokyo, Japan Hospital, Tokyo, Japan

*Drs Fukuma, Tokiwa and Numata contributed equally to this work

Correspondence to

Eiki Takimoto, M.D. Ph.D.

Department of Cardiovascular Medicine, Graduate School of Medicine, The University of Tokyo

7-3-1, Hongo, Bunkyo, Tokyo, Japan 113-8655

E-mail: etakimoto-tky@umin.ac.jp

Tel: +81 (03) 3815-5411, Fax: +81 (03) 5800-9171

Sup Fig. 1A

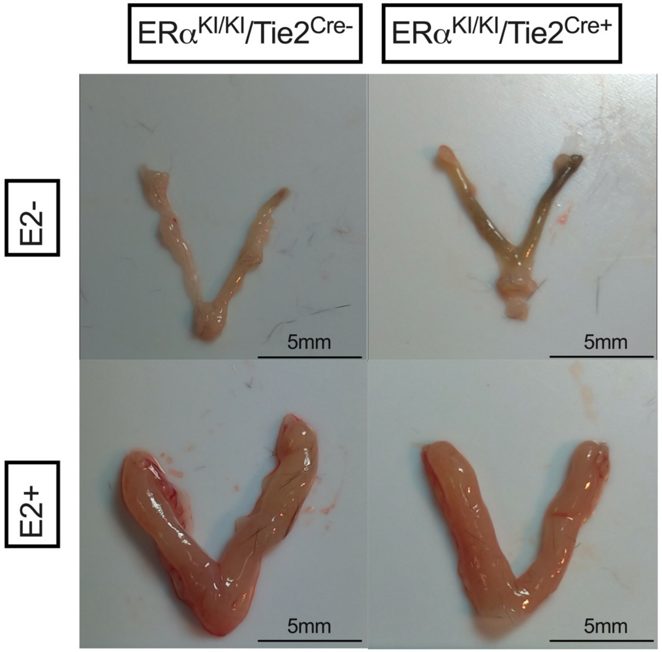

Sup Fig. 1B

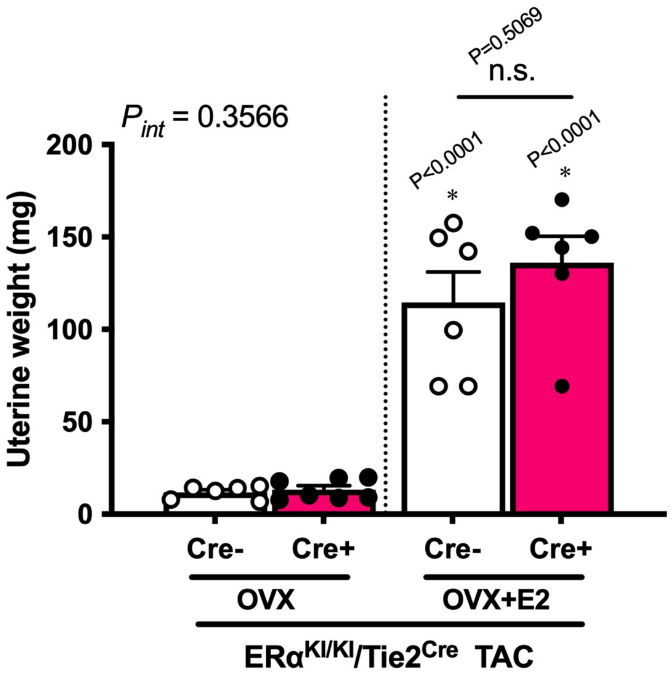

Supplementary Fig. 1 Uterine morphology and weight of ERα$^{KI/KI}$/Tie2^{Cre} mice after OVX with or without E2 supplementation

A. Representative uterine morphology

B. Uterine weight (mg) of ERα$^{KI/KI}$/Tie2^{Cre} mice (n = 6–7 per group); $^{*}P < 0.05$ vs OVX was determined by Tukey's HSD test following 2-way ANOVA; P_{int}, interaction P value as determined by 2-way ANOVA; Scatter dot plots with bars show individual values and mean ± SEM; abbreviations are as in Figure 1 and 2

Sup Fig. 2A

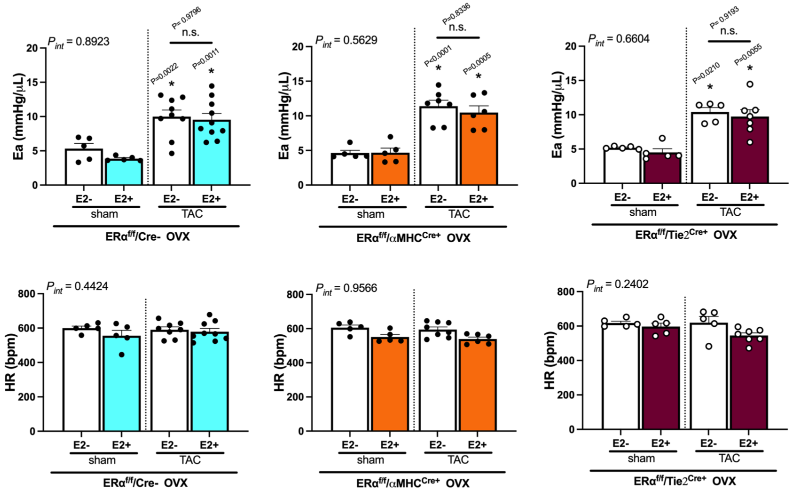

Sup Fig. 2B

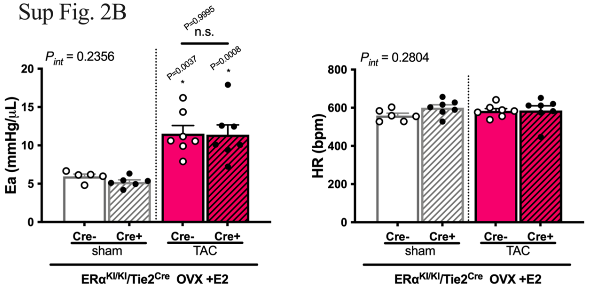

Supplementary Fig. 2 Parameters from PV loop analyses

A. Ea (mmHg/μL) and HR (bpm) of sham or 3-week TAC hearts in ERα$^{f/f}$/Cre-, ERα$^{f/f}$/αMHC^{Cre+} and ERα$^{f/f}$/Tie2^{Cre+} mice (n = 5–10 per group)

B. Ea (mmHg/μL) and HR (bpm) of sham or 3-week TAC hearts in ERα$^{KI/KI}$/Tie2^{Cre-} and ERα$^{KI/KI}$/Tie2^{Cre+} mice (n = 5–7 per group); $^{*}P < 0.05$ vs sham were determined by Tukey's HSD test following 2-way ANOVA; P_{int}, interaction P value as determined by 2-way ANOVA; Scatter dot plots with bars show individual values and mean ± SEM; Ea: effective arterial elastance; HR: heart rate; other abbreviations are as in Figure 1 and 2

Sup Fig. 3A

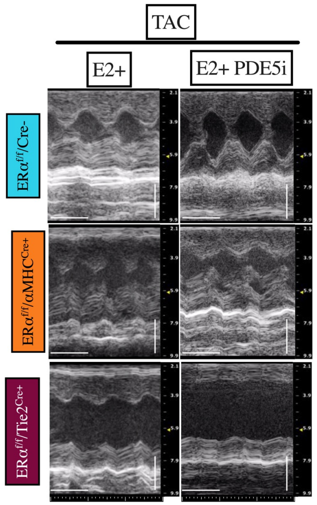

Sup Fig. 3B

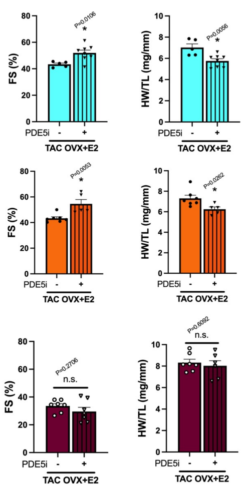

Sup Fig. 3C

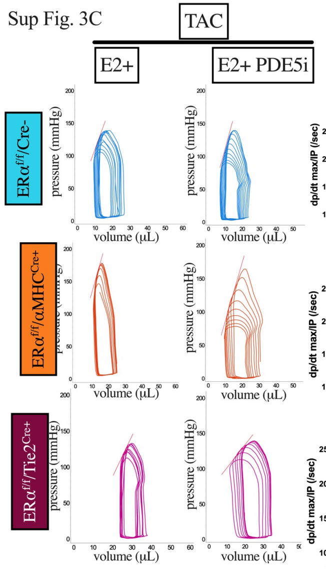

Sup Fig. 3D

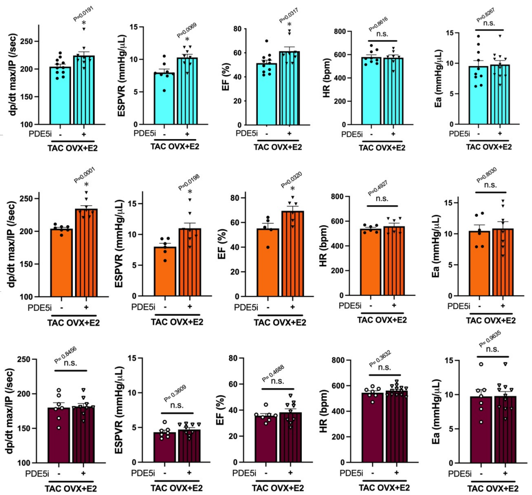

Supplementary Fig. 3 Cardiac phenotype of tissue-specific ERα-knockout mice during pressure-overload with PDE5i in the presence of E2

A. Representative M mode images of LV by echocardiography at 3 weeks after TAC surgery, time stamp: 100 msec, vertical bar: 2 mm

B. Effect of PDE5i on LV FS (%) and HW (mg) normalized to TL (mm) at 3 weeks after TAC surgery (n = 5–8 per group)

C. Representative PV loops of ERα$^{f/f}$/Cre-, ERα$^{f/f}$/αMHC^{Cre+} and ERα$^{f/f}$/Tie2^{Cre+} hearts subjected to pressure-overload for 3 weeks. Loops during preload reduction and ESPVR (upper left straight lines) are shown

D. dP/dt max/IP (/sec), ESPVR slope (mmHg/mL), EF (%), HR (bpm) and Ea (mmHg/μL) in each group (n = 5–13 per group); $^{*}P < 0.05$ was determined by Student's t-test; Scatter dot plots with bars show individual values and mean ± SEM PDE5i: phosphodiesterase-5 inhibitor; other abbreviations are as in Figure 1, 2 and Supplementary Fig. 2

Sup Fig. 4

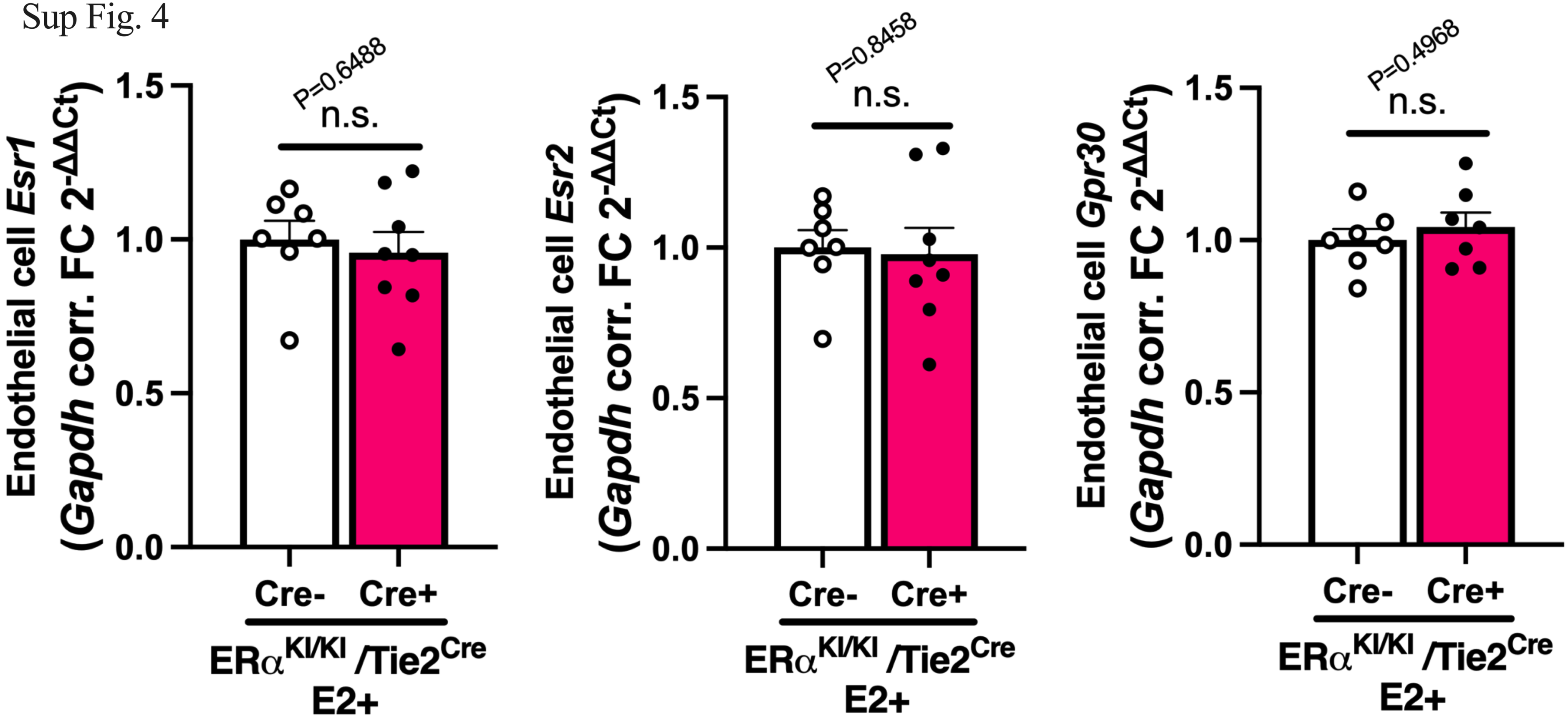

Supplementary Fig. 4 Gene expressions of estrogen receptor subtypes in ERα$^{KI/KI}$/Tie2^{Cre} ECs
Esr1, *Esr2* and *Gpr30* mRNA expressions in isolated ECs normalized to *Gapdh* (n = 7–9 per group); Statistical significancy was assessed using Student's t-test; Scatter dot plots with bars show individual values and mean ± SEM; abbreviations are as in Figure 1 and 7

Sup Fig. 5A

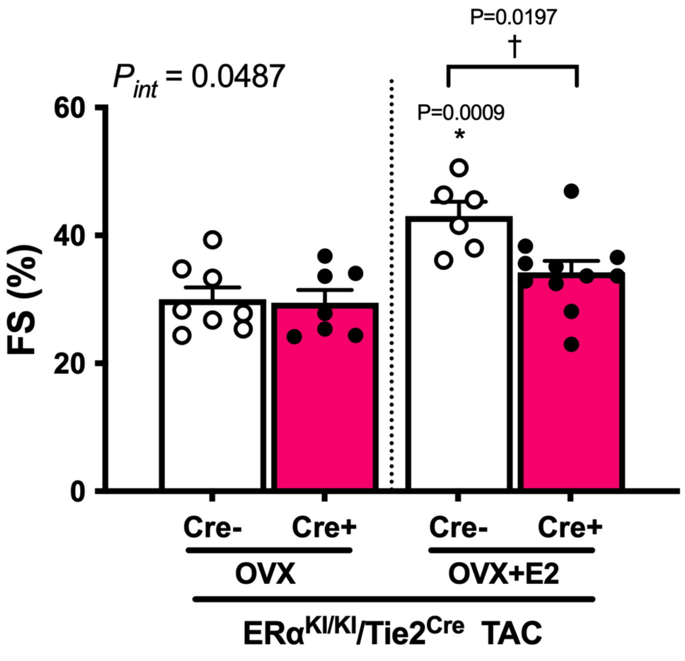

Sup Fig. 5B

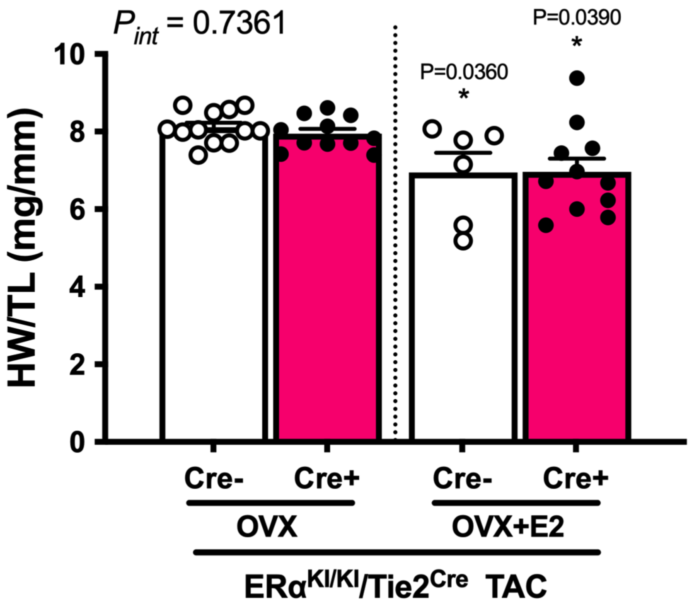

Supplementary Fig. 5 Comparison of cardiac phenotype between OVX and OVX+E2 in ERα$^{KI/KI}$/Tie2^{Cre} mice subjected to 3-week TAC

A. LV FS (%) comparison at 3 weeks after TAC surgery (n = 6–11 per group)

B. Heart weight (HW) (mg) normalized to tibial length (TL) (mm) at 3 weeks after TAC surgery (n = 6–12 per group); $^{*}P < 0.05$ vs OVX and $^{\dagger}P < 0.05$ were determined by Tukey's HSD test following 2-way ANOVA; P_{int}, interaction P value as determined by 2-way ANOVA; Scatter dot plots with bars show individual values and mean ± SEM; abbreviations are as in Figure 1 and 2

Sup Fig. 6A

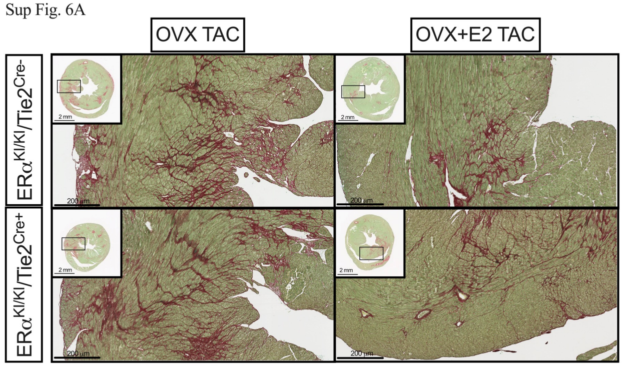

Sup Fig. 6B

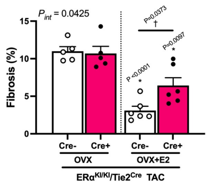

Sup Fig. 6C

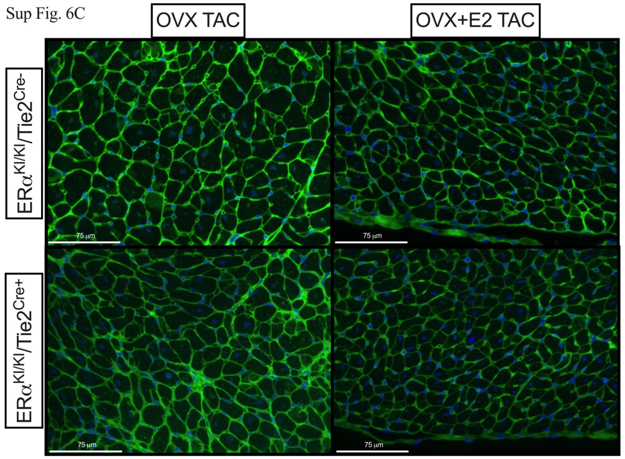

Sup Fig. 6D

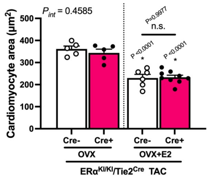

Supplementary Fig. 6 Histological assessments for LV fibrosis and LV cardiomyocyte area in ERα$^{KI/KI}$/Tie2^{Cre} hearts subjected to pressure-overload for 3 weeks

A. Representative images of LV sections stained with Picrosirius red and Fast-green at 3 weeks after TAC surgery (scale bars: 2 mm and 200 μm)

B. Quantification results for LV fibrosis (%) at 3 weeks after TAC surgery (n = 5-9 per group)

C. Representative images of LV sections stained with Alexa Fluor 488-conjugated wheat germ agglutinin (WGA) and DAPI at 3 weeks after TAC surgery (scale bar: 75 μm)

D. Quantification results of cardiomyocyte area (μm^2) at 3 weeks after TAC surgery (n = 5-9 per group); *P < 0.05 vs OVX and †P < 0.05 were determined by Tukey's HSD test following 2-way ANOVA; P_{int}, interaction P value as determined by 2-way ANOVA; Scatter dot plots with bars show individual values and mean ± SEM; abbreviations are as in Figure 1 and 2

Sup Fig. 7A

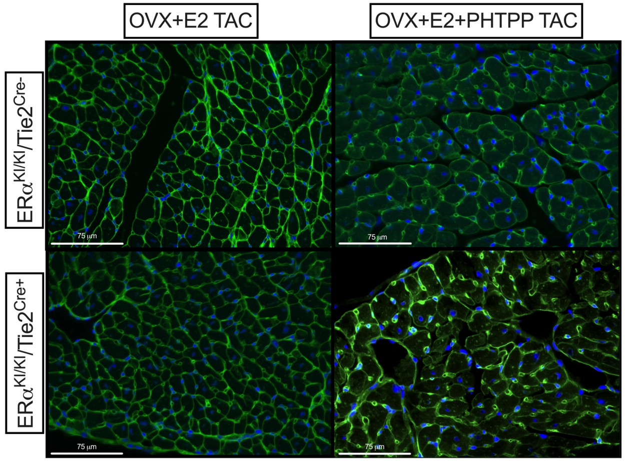

Sup Fig. 7B

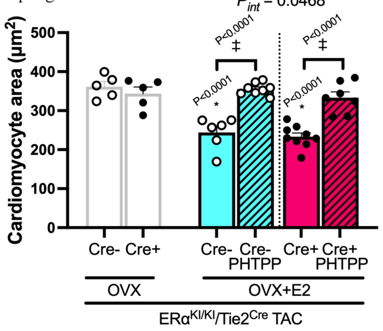

Sup Fig. 7C

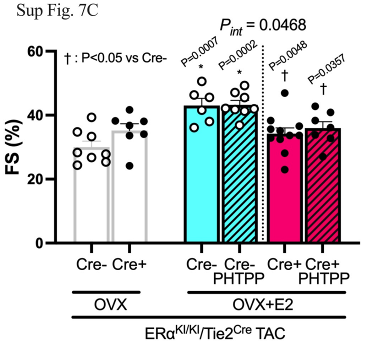

Supplementary Fig. 7 Effects of ERβ antagonist on LV cardiomyocyte area and cardiac function in ERα$^{KI/KI}$/Tie2^{Cre} hearts subjected to pressure-overload for 3 weeks

A. Representative images of LV sections stained with Alexa Fluor 488-conjugated wheat germ agglutinin (WGA) and DAPI at 3 weeks after TAC surgery (scale bar: 75 µm)

B. Quantification results of cardiomyocyte area (µm^2) at 3 weeks after TAC surgery, shown with OVX+TAC surgery groups (n = 5-9 per group)

C. LV FS (%) at 3 weeks after TAC surgery, shown with OVX+TAC surgery groups (n = 6–11 per group); $^*P < 0.05$ vs OVX was determined by Tukey's HSD test following 1-way ANOVA. $^{†}P < 0.05$ vs Cre- and $^{‡}P < 0.05$ were determined by Tukey's HSD test following 2-way ANOVA in OVX+E2 groups; P_{int}, interaction P value as determined by 2-way ANOVA in OVX+E2 groups; Scatter dot plots with bars show individual values and mean ± SEM; PHTPP: ERβ-specific antagonist; other abbreviations are as in Figure 1 and 2

Sup Fig. 8A

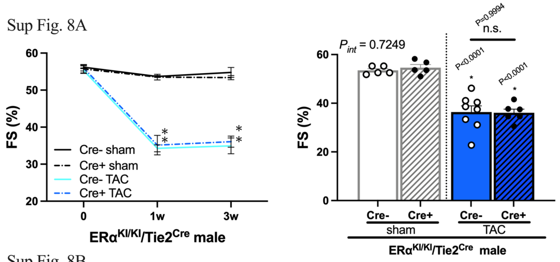

Sup Fig. 8B

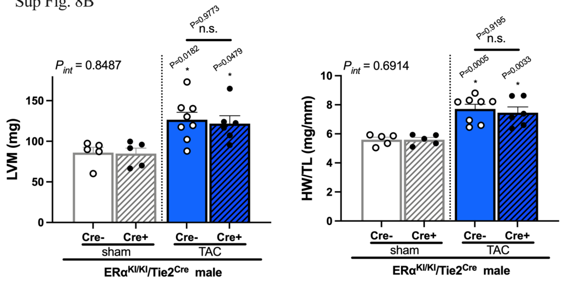

Sup Fig. 8C

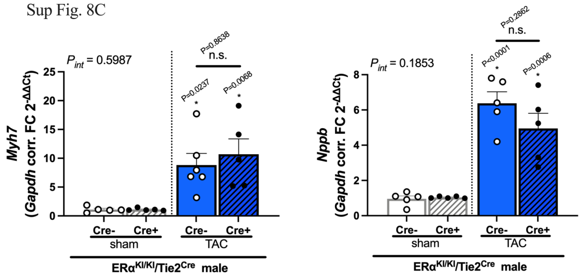

Supplementary Fig. 8 Cardiac phenotype of ERα$^{KI/KI}$/Tie2^{Cre} male exposed to LV pressure-overload for 3 weeks

A. LV FS (%) time course (pre, 1week and 3week; left panel) and LV FS (%) comparison at 3 weeks after TAC surgery (right panel) (n = 5–8 per group)

B. Left ventricular mass (LVM) (mg) by echocardiography (upper panel) and post-mortal heart weight (HW) (mg) normalized to tibial length (TL) (mm) (lower panel) at 3 weeks after TAC surgery (n = 5–8 per group)

C. Gene expression in the left ventricular myocardium at 3 weeks after TAC surgery normalized to *Gapdh* (n = 5–6 per group); $^{*}P < 0.05$ vs sham was determined by Tukey's HSD test following 2-way ANOVA; P_{int}, interaction P value as determined by 2-way ANOVA; Scatter dot plots with bars show individual values and mean ± SEM; abbreviations are as in Figure 1 and 2

Supplementary table 1: Primer sequences

bmhc(Myh7)	forward	ATGTGCCGGACCTTGGAAG
	reverse	ATGTGCCGGACCTTGGAAG
Atp2a2	forward	TCGACCAGTCAATTCTTACAGG
	reverse	CAGGGACAGGGTCAGTATGC
Vegfa	forward	GTACCTCCACCATGCCAAGT
	reverse	TCATGGGACTTCTGCTCTCC
Nppb	forward	AAGTCCTAGCCAGTCTCCAGA
	reverse	GAGCTGTCTCTGGGCCATTTC
Gapdh	forward	CATGGCCTTCCGTGTTCCTA
	reverse	CCTGCTTCACCACCTTCTTGAT
Il1b	forward	AGTTGACGGACCCCAAAAG
	reverse	AGCTGGATGCTCTCATCAGG
Il6	forward	CTGCAAGAGACTTCCATCCAG
	reverse	AGTGGTATAGACAGGTCTGTTGG
Tgfb	forward	TGACGTCACTGGAGTTGTACGG
	reverse	GGTTCATGTCATGGATGGTGC
Ppargc1a	forward	ATAGAGTGTGCTGCTCTGGTTGGT
	reverse	TGGTCGCTACACCACTTCAATCCA
Esr1	forward	TCTGCCAAGGAGACTCGCTACT
	reverse	GGTGCATTGGTTTGTAGCTGGAC
Esr2	forward	GGTCCTGTGAAGGATGTAAGGC
	reverse	TAACACTTGCGAAGTCGGCAGG
Gpr30	forward	GCCACATAGTCAACCTTGCAGC
	reverse	CGTCTTCTGCTCCACATAGAGC
